# A VIM2/4 deletion, and premature truncations of *CMT2* and *FBX5*, drive DNA methylation changes after colonization of a novel habitat

**DOI:** 10.1038/s41477-026-02352-2

**Published:** 2026-08-03

**Authors:** Johan Zicola, Emmanuel Tergemina, Ahmed F. Elfarargi, Mehmet Göktay, Célia Neto, Robert J. Schmitz, Angela M. Hancock

**Affiliations:** 1 Max Planck Institute for Plant Breeding Research, Cologne, Germany; 2 Department of Crop Sciences, Center for Integrated Breeding Research, Georg-August-University, Göttingen, Germany; 3 Department of Integrative Biology, The University of Texas at Austin, Austin, TX USA; 4 Department of Plant and Environmental Sciences, University of Copenhagen, Copenhagen, Denmark; 5 Department of Genetics, University of Georgia, Athens, GA USA; 6 Department of Botany and Plant Pathology, Purdue University, West Lafayette, IN USA; 7 Center for Plant Biology, Purdue University, West Lafayette, IN USA

**Keywords:** Natural variation in plants, Epigenomics

## Abstract

DNA methylation is important to maintain genome stability, but alterations in genome-wide methylation patterns can produce widespread genomic effects, with the potential to facilitate rapid adaptation. Here we investigate DNA methylation evolution in *Arabidopsis thaliana* during its colonization of the drought-prone Cape Verde Islands (CVI). We identified three high-impact changes in genes linking histone modification to DNA methylation that underlie variation in DNA methylation within CVI. We show that gene body methylation is reduced in CVI relative to the Moroccan outgroup due to a 2.7-kb deletion between two *VARIANT IN METHYLATION*genes (*VIM2*and *VIM4*), causing aberrant expression of the *VIM2/4* homologues. Disruptions of *CHROMOMETHYLASE 2* (*CMT2*) and a newly identified DNA methylation modulator, *F-BOX PROTEIN 5* (*FBX5*), which we validated using CRISPR mutant analysis, contribute to DNA methylation of transposable elements within CVI. Overall, our results reveal rapid methylome evolution driven largely by high-impact variants in three genes.

## Abstract (web summary)

This study shows that *Arabidopsis thaliana* colonizing the Cape Verde Islands rapidly evolved altered DNA methylation patterns through high-impact mutations affecting *VIM2/4*, *CMT2* and *FBX5*, linked to alternative drought adaptation strategies.

## Main

Epigenetic modifications of DNA, primarily 5-methylcytosine, are crucial for maintaining genome stability^1–4^. While methylation in mammals occurs primarily at CG dinucleotides, plants exhibit methylation in all cytosine contexts: symmetric CG and CHG, and asymmetric CHH (where H is C, A or T). DNA methylation serves multiple functions, including silencing transposable elements (TEs), regulating gene expression, processing RNA and mediating chromosome interactions^4,5^.

In plants, distinct methyltransferases govern different methylation contexts: METHYLTRANSFERASE 1 (MET1) maintains CG methylation, while CHROMOMETHYLASE 3 (CMT3) maintains CHG methylation^6–12^. CHH methylation is established by DOMAIN REARRANGED METHYLTRANSFERASE 1/2 (DRM1/2) through the RNA-directed DNA methylation pathway^13,14^, whereas CHROMOMETHYLASE 2 (CMT2) acts in heterochromatic regions independently of small RNA activity^12,15^. A feedback loop exists between histone modifications and DNA methylation, exemplified by CMT3’s dependence on histone 3 lysine 9 dimethylation (H3K9me2) deposited by SU(VAR)3-9 HOMOLOG 4 (SUVH4) (also called KRYPTONITE)^8,9,11,16–19^. In addition, small euchromatic TEs are targeted by RNA-directed DNA methylation through small interfering RNAs and maintained by DRM2, while methylation of long heterochromatic TEs requires DECREASED DNA METHYLATION 1 (DDM1) in conjunction with CMT2^15^.

In *Arabidopsis thaliana* (referred to as *Arabidopsis* throughout), genome-wide loss of DNA methylation affects gene expression and TE repression but is not lethal^10,15,20,21^. While CG-dinucleotide methylation of promoters typically suppresses transcription^22^, it is relatively rare in plants^23^. Gene body methylation (gbM), predominately in the CG context, occurs in approximately 20% of *Arabidopsis* genes and correlates with moderate and constitutive expression^24–34^. GbM is maintained by MET1 and probably also requires CMT3 for its initiation and maintenance since plant species without functional *CMT3* lack gbM^35,36^. Despite ongoing debate about whether gbM is merely a by-product of TE silencing^35,37,38^, or serves functions such as gene expression stabilization, cryptic transcription inhibition and protection against TE insertion^39,40^, conservation across diverse plant species suggests functional importance^30,39,41,42^.

Studying natural variation in DNA methylation can provide insights into how epigenetic changes evolve over time in natural populations and connect to other physiological and molecular changes^31,43–45^. Previous studies of natural variation in gbM among *Arabidopsis* accessions identified Cvi-0, from the Cape Verde archipelago, as having the lowest gbM levels^43,45–47^. Historical reconstructions indicate that *Arabidopsis* colonized this drought-prone region approximately 5,000 years ago from Morocco through a strong bottleneck that eliminated most standing genetic variation, necessitating rapid adaptation through new variants^48^.

Here, we investigate DNA methylation patterns in the Cape Verde Islands (CVI) population, hypothesizing that mutations affecting genome-wide epigenetic profiles could influence many genes simultaneously, facilitating rapid adaptation. We find mean gbM is reduced in this population compared with both the closest Moroccan outgroup and well-studied Eurasian populations. Through genome-wide association studies (GWASs) of methylation signatures across different cytosine contexts, we identify novel alleles in known methylation modulators (VIM2/4 and CMT2) and discover a previously uncharacterized methylation regulator (FBX5), with effects confirmed through CRISPR mutant analysis. We examine the evolutionary history and links to physiological traits of these variants and find they are associated with drought adaptation strategies.

## Results

### Gene body methylation is reduced in *Arabidopsis* from Cape Verde

**Fig. 1:**
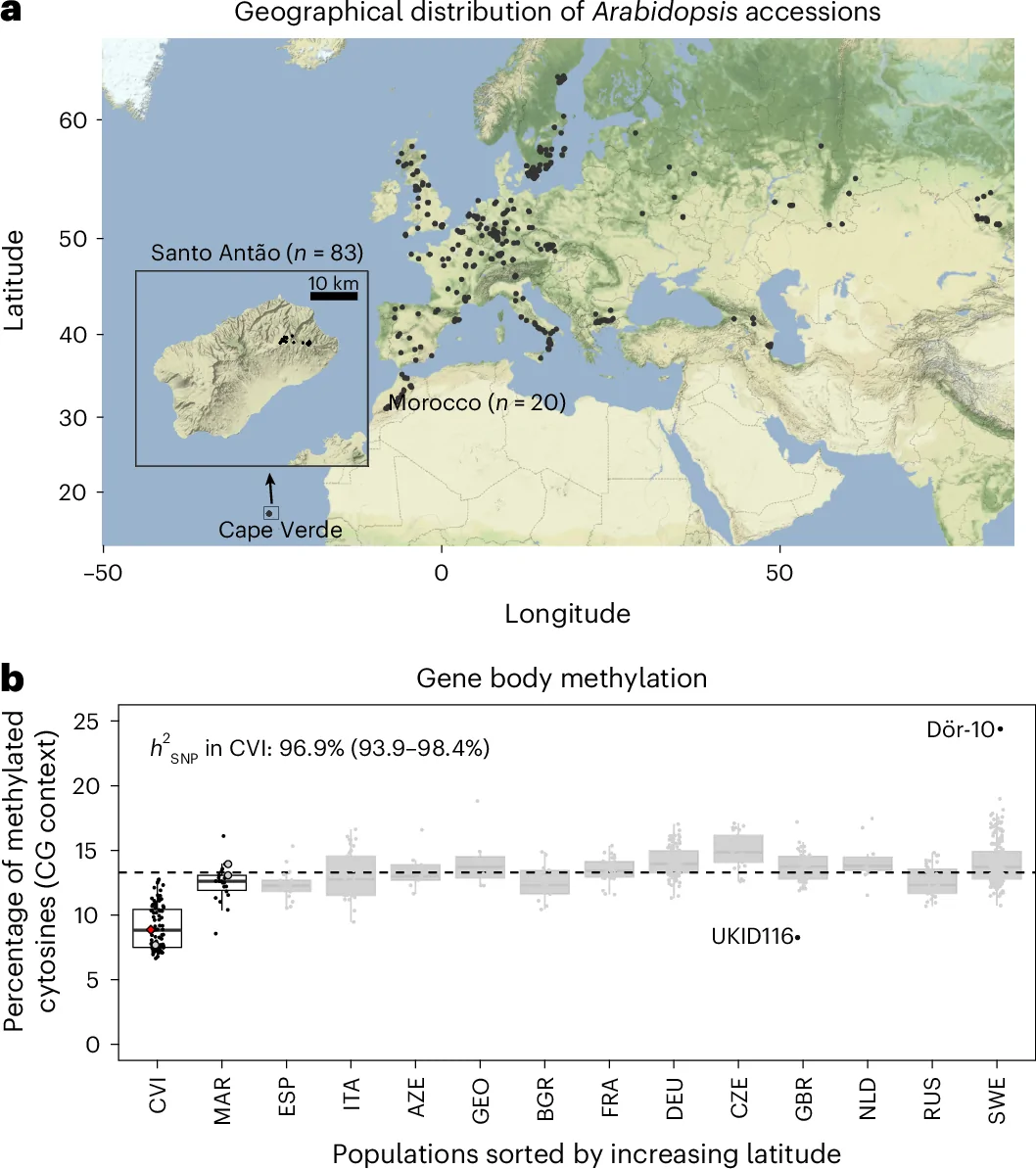
*Arabidopsis* populations from CVI diverge epigenetically from all other known populations. **a**, The geographical distribution of the bisulfite-sequenced accessions from the 1001GP and this study. Only countries with at least eight bisulfite-sequenced accessions are shown. New data were collected in this study for CVI and Morocco, indicated on the map and the inset. **b**, Gene body methylation for the CG context for the accessions shown in **a**, grouped by country (three-letter codes are the ISO names, except CVI; MAR indicates Morocco), and ordered by increasing latitude. The centre lines of box plots show the medians, boxes denote the 25th and 75th percentiles, whiskers extend 1.5 times the interquartile range from the 25th and 75th percentiles and dots represent individual data points. Dashed lines indicate the median values across all countries. Grey box plots represent methylation levels from published data (representative subset of 523 accessions of the 1001GP). The grey circles indicate methylation levels for published data for Cvi-0 and MAR (two accessions). The red diamond indicates the methylation level of Cvi-0 from our data. The highlighted accessions UKID116 (GBR) and Dör-10 (SWE) were previously identified as having the lowest and highest gbM levels worldwide, respectively^45^. SNP heritability (*h*^2^_snp_) for gbM in CVI and its 95% CI are indicated on the plot. Basemap data in **a** from Stadia Maps, OpenMapTiles and OpenStreetMap.

We assessed gbM variation in the Santo Antão population of 83 accessions from CVI using whole-genome bisulfite sequencing. For comparison, we also conducted whole-genome bisulfite sequencing on 22 representative accessions from Morocco, the closest outgroup population to CVI^48^ (Fig. 1a and Supplementary Table 1**)** and contrasted these to published whole-genome bisulfite sequencing data from Eurasian samples^45^ (Fig. 1a and Supplementary Table 2). Average gbM levels of CVI plants were significantly lower than any other population (Wilcoxon test, *P* value <2.2 × 10^−16^), including the Moroccan outgroup (Wilcoxon test, *P* = 2.6 × 10^−11^), which exhibits gbM levels similar to other worldwide populations (Fig. 1b). We estimated single-nucleotide polymorphism (SNP)-based heritability^49^ of gbM levels in the CVI population to be 97% based on the correlation between phenotypic and genetic covariance in a Bayesian sparse linear mixed model (LMM) that accounts for small and large effect variants^50^.

### A 2.7-kb deletion of the *VIM2* and *VIM4* promoters explains the reduced gene body methylation in Cape Verde

**Fig. 2:**
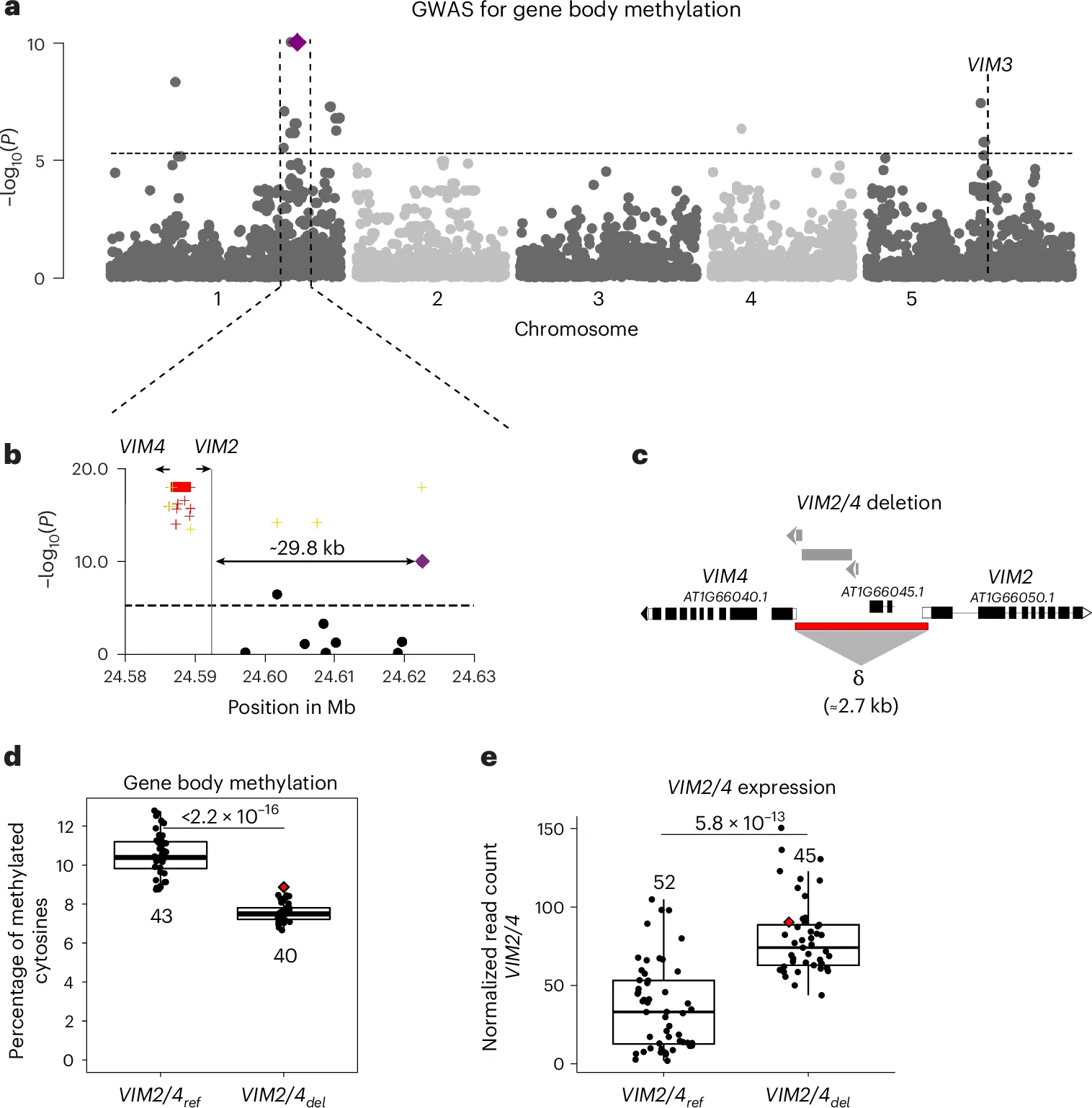
A 2.7-kb deletion causes the loss of the VIM2 and VIM4 promoters and is associated with low gbM. **a**, Manhattan plot showing results of GWAS of gbM in CVI. The 10,321 SNPs with a MAF of at least 5% were included in the analysis. The Bonferroni threshold is indicated by the dashed line. **b**, *k*-mer GWAS reveals a 2.7 kb deletion between VIM2 and VIM4. *k*-mers are highlighted in yellow if they mapped to the Cvi-0 assembly and in red if they did not map to the Cvi-0 assembly. SNPs are highlighted in black. The most significant SNP is annotated with a purple diamond. **c**, The deletion at VIM2 and VIM4 (red box) overlaps the 5′-UTRs (white boxes) of both genes but does not affect the start codons. Grey boxes indicate the locations of TEs. **d**, Difference in gbM between the accession without (0) and with the VIM2/4 deletion (1). A two-tailed Welch’s *t*-test was performed to compare the means of the groups and the *P* value is shown in the plot. **e**, The expression differential between the two VIM2/4 alleles is shown with normalized read counts in 97 accessions from CVI. A two-tailed *t*-test was performed between the two groups and the *P* value is shown in the plot. In **d** and **e**, the centre lines of box plots show the medians, box limits the 25th and 75th percentiles, whiskers extend 1.5 times the interquartile range from the 25th and 75th percentiles and dots represent individual data points. The red diamond represents gbM for Cvi-0. The number of individuals for each group is indicated below each box. *P* values are indicated on the plots.

To identify genetic modifiers of gbM in the CVI population, we conducted a GWAS of DNA methylome data using per cent DNA methylation over gene bodies as a phenotype. We identified a major peak on chromosome 1 (Fig. 2a), although no SNP in that genomic region localized to a gene known to be involved in DNA methylation. This could occur if the peak was caused by an untyped genetic variant, such as a structural variant. To investigate the possibility that structural variation was responsible for the peak, we examined associations with *k*-mers^51^. To localize *k*-mers to the genome, we aligned significantly associated *k*-mers to the Col-0 reference assembly as well as to four newly created long read-based genome assemblies from CVI accessions (S1-1, S15-3, S5-10 and Cvi-0) (Supplementary Fig. 1a). A group of 1,657 of the most significant *k*-mers flanked the promoter regions of *VARIANT IN METHYLATION 2* (*VIM2*, AT1G66050) and *VIM4* (AT1G66040), which are positioned head-to-head approximately 2.7 kb apart in Col-0 (Fig. 2b). *VIM2* and *VIM4* are excellent candidate genes because they are involved in CG methylation maintenance^52,53^. These *k*-mers were mappable in the S1-1 and S15-3 accessions, but unmappable in the Cvi-0 and S5-10 assemblies, consistent with a segregating deletion in the *VIM2/4* promoter region in Cape Verde.

**Extended Data Fig. 1:**
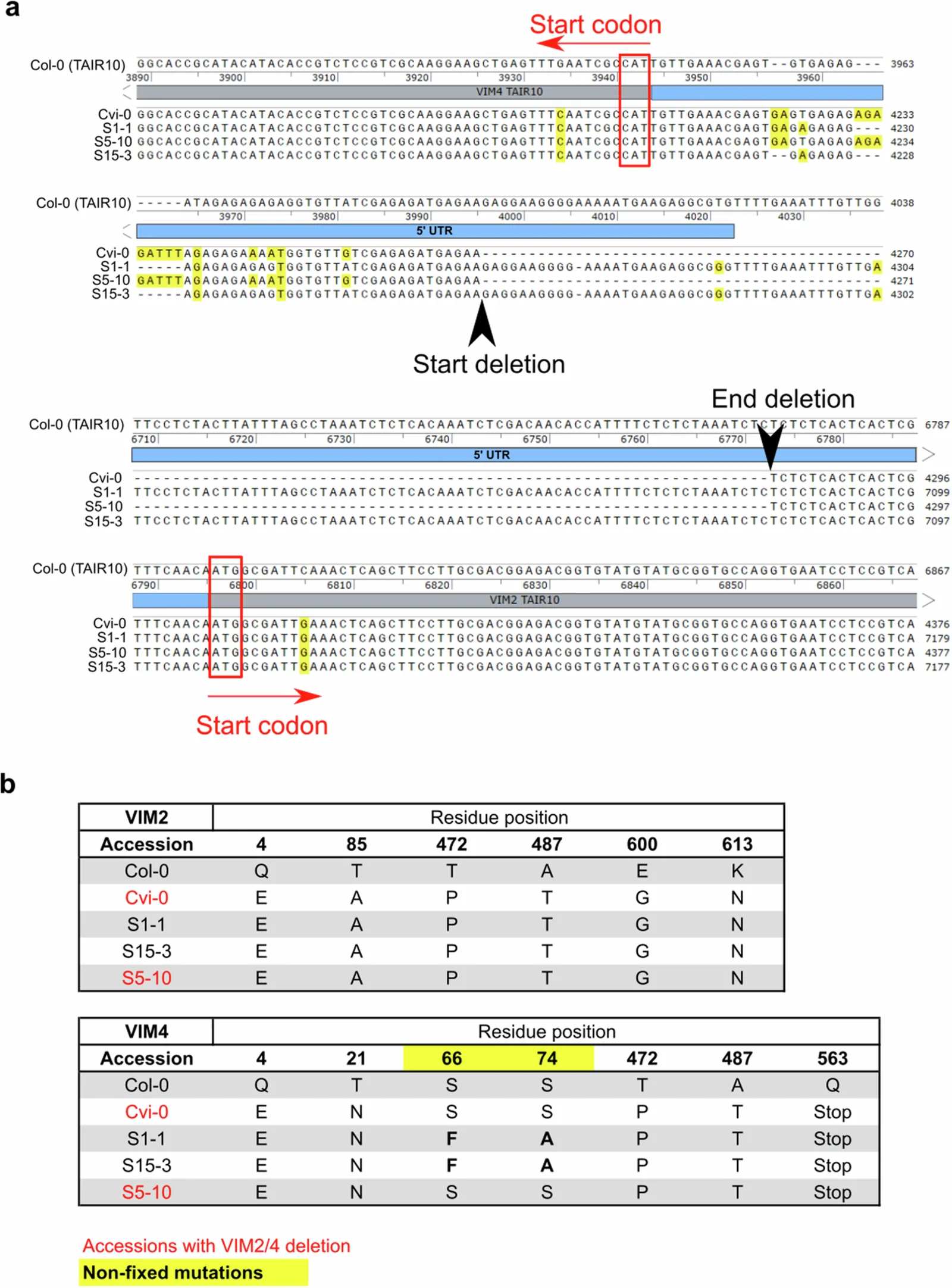
VIM2/4 deletion and polymorphisms at VIM2 and VIM4. **a**, DNA sequence alignment at the *VIM2/4* region using TAIR10 Col-0 assembly and the four *de novo* assemblies generated for Cvi-0, S1-1, S5-10, and S15-3. Yellow highlights indicated polymorphisms compared to Col-0, black arrows indicate the position of the deletion. Start codons of *VIM4* (bottom strand) and *VIM2* (top strands) are indicated with a red arrow. 5’ UTRs are indicated in blue and CDSs in gray. **b**, Polymorphisms leading to non-synonymous substitutions or premature stop codons at *VIM2* and *VIM4* within the four CVI accessions with long-read genome assemblies. Accessions in red contain the *VIM2/4del* variant and residue positions highlighted in yellow are segregating across CVI.

By comparing the *VIM2/4* genomic region of the two CVI accessions carrying the deletion with the Col-0 reference assembly, we found that the start codons of the two *VIM* genes are preserved, but a part of the 5′ untranslated region (UTR) is removed for each gene, which could impact the regulation of *VIM2*/*VIM4* (Fig. 2c and Extended Data Fig. 1a**)**. The deletion contains three RC/helitron fragments and a reverse transcriptase-like gene (AT1G66045) in Col-0, suggesting that the deletion may have resulted from a transposition event. We also found that the CVI population carries a premature stop codon at amino acid position 563 of VIM4, indicating that *VIM4* is probably nonfunctional in CVI (Extended Data Fig. 1b). In addition to the peak at *VIM2/4*, we identified a genome-wide significant signal colocalizing with *VIM3*. However, the top SNP lies 790 kb upstream of *VIM3* in Col-0, within a TE (AT5TE54335) located in the fourth exon of a NOD26-like intrinsic protein (AT5G37810), shows poor linkage disequilibrium with the surrounding SNPs and is in complete linkage disequilibrium (*D*′ = 1) with the *VIM2/4* deletion, suggesting it is an artefact (Supplementary Fig. 1b). To explore whether there was any evidence of a structural variant affecting VIM3 function, we also examined the kmerGWAS results and identified a *k-*mer mapping 93 bp upstream of *VIM3* (Supplementary Fig. 1c,d). However, while this *k*-mer maps with highest affinity to the *VIM3* promoter region in Col-0, in CVI it maps with the highest affinity to the *VIM2/4* region and with lower affinity to *VIM3*. In CVI, it maps without any mismatches to the *VIM2/4* intergenic region in S1-1 and S15-3 (*VIM2/4*_ref_), and with one mismatch on chr5 93-bp upstream of *VIM3*. We conclude that the GWAS signals near *VIM3* on chromosome 5 are most probably due to a mapping artefact and are driven by the *VIM2/4* deletion rather than variation on chromosome 5.

We genotyped the *VIM2/4* deletion in the CVI population and found that mean gbM levels were reduced by 27.9% for accessions carrying the *VIM2/4* deletion (*VIM2/4*_del_) relative to the reference allele (*VIM2/4*_ref_), corresponding to an absolute gbM reduction of 2.94% methylation in the LMM (Fig. 2d). *VIM2/4*_del_ is found in 20 of the 24 sites where *Arabidopsis* was collected across Santo Antão, with an overall frequency of 47% (89/189) on the island (Supplementary Fig. 2). Using *VIM2/4*_del_ as a covariate, no other regions were significantly associated with variation in gbM, indicating again that *VIM2/4*_del_ is sufficient to explain the variation in the chromosome 5 region (Supplementary Fig. 3).

To assess whether promoter deletions of *VIM2* could have occurred independently outside of CVI, we screened for low read coverage in the region in the accessions from the 1001GP. We identified six accessions in addition to Cvi-0 with low read counts mapping to the VIM2/4 promoter region: three accessions from Spain (seqIDs 9571, 9822 and 9854), two accessions from Sweden (seqIDs 6124 and 6231) and one accession from Finland (seqID 7126). These findings support the hypothesis that the region is unstable due to the helitrons located within the region; however, in contrast to CVI, these other deletions are not present at high frequency in European populations.

To assess the potential impacts of *VIM2/4*_del_ more broadly, we examined which regions were differentially methylated between accessions with the *VIM2/4*_ref_ and the *VIM2/4*_del_ allele. Using a threshold of 25% difference in methylated CG sites (mCG), we identified 12,996 differentially methylated regions (DMRs) with 94% showing lower mCG levels in the accessions with *VIM2/4*_del_ (Supplementary Table 3). Most of the DMRs (92%, *n* = 11,948) overlap genic regions (*n* = 7,541), while 8% (*n* = 978) overlap TEs. Out of the 27,655 protein-coding genes from the Araport11 annotation, 26% (*n* = 7,200) co-localize with at least one DMR (Supplementary Table 4).

**Extended Data Fig. 2:**
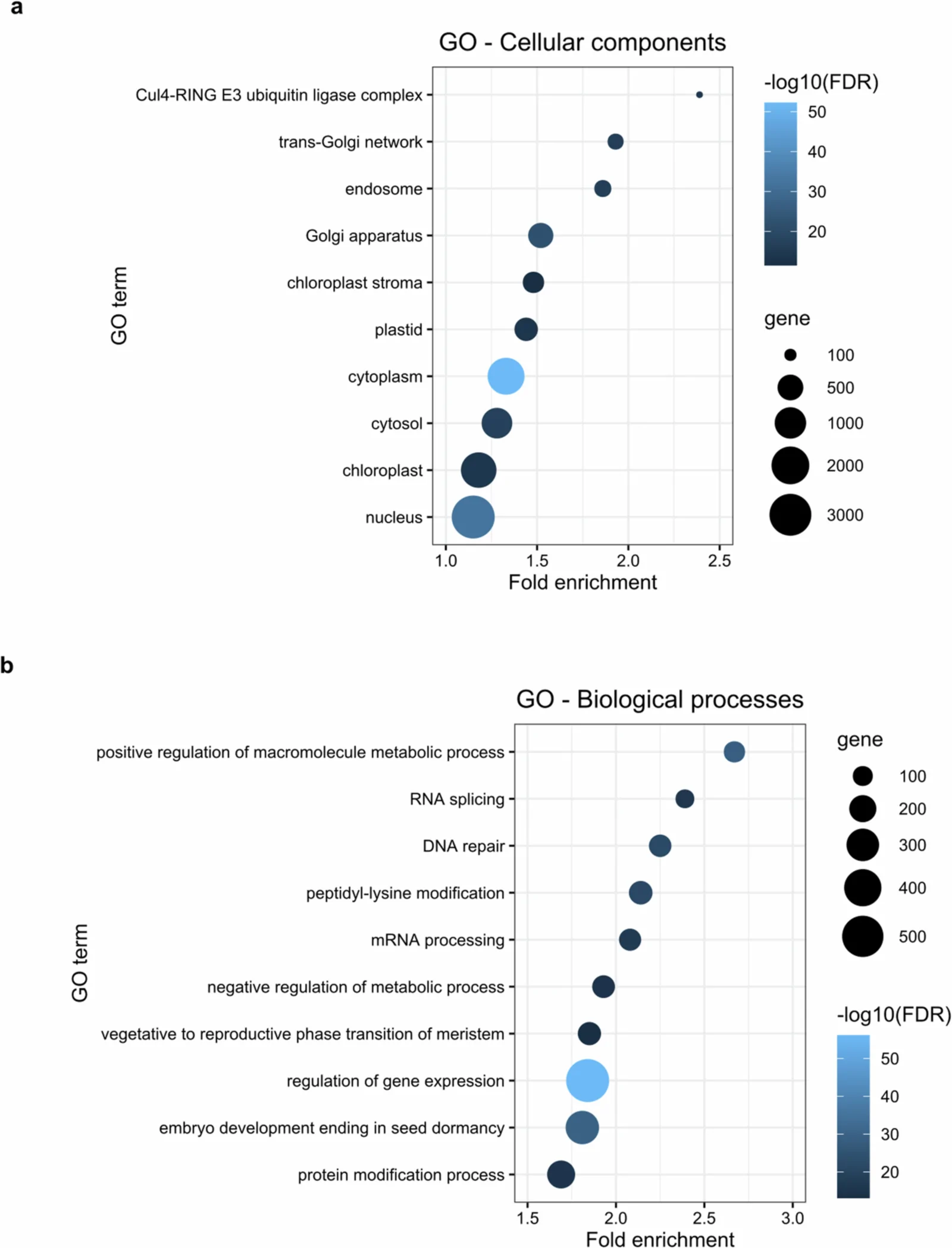
GO enrichment analysis at VIM2 mCG DMRs. Fold enrichment of the ten most significant terms for cellular components (CC) (**a**) and biological processes (BP) (**b**) for 7,200 genes overlapping mCG DMRs. GO terms are displayed by decreasing Fold enrichment from top to bottom. Full GO enrichment results are in Supplementary Table 5.

Gene Ontology (GO) enrichment analysis on protein-coding genes overlapping DMRs revealed a strong enrichment for the cellular components ontology GO term Cul4–RING E3 ubiquitin ligase complex (GO:0080008; 2.4-fold, Benjamini-corrected *P* value of 2 × 10^−15^), which was driven by 79 genes, many of which are known to be involved in environmental response traits, including light signalling, abscisic acid signalling, timing of flowering and DNA repair (Extended Data Fig. 2a,b and Supplementary Table 5). Biological processes enriched for DMRs included those directly related to expected large-scale effects of DNA methylation, such as regulation of gene expression (1.8-fold, Benjamini-corrected *P* value of 8 × 10^−57^) and chromatin remodelling (2.6-fold, Benjamini-corrected *P* value of 5.9 × 10^−10^), as well as biological processes related to DNA repair (2.3-fold, Benjamini-corrected *P* value of 2.4 × 10^−22^) and embryo development ending in seed dormancy (1.8-fold, Benjamini-corrected *P* value of 2.4 × 10^−30^) (Extended Data Fig. 2a,b and Supplementary Table 6).

Leaf transcriptomes clustered mainly by population, which is expected if transcriptome variation is due to genetic factors (Supplementary Fig. 4 and Supplementary Table 7**)**. Ninety-four genes were significantly differentially expressed between the *VIM2/4*_del_ and *VIM2/4*_ref_ genotypes, including several transposable element-derived genes and genes involved in abscisic acid response and chloroplast function (Supplementary Table 8). Contrary to our expectations, expression of *VIM2* and *VIM4* was increased in *VIM2/4*_del_ accessions. While we expected the loss of promoter regions in *VIM2/4*_del_ accessions would abolish *VIM2* and *VIM4* expression, we instead found that the genes were upregulated when the UTRs were deleted, suggesting they still have functional minimal promoters and the UTR may contain inhibitory *cis*-regulatory elements or otherwise result in a stronger promoter (Fig. 2e). We note that the DNA methylation profile we measured in rosette leaves may not be representative of the expression profiles in other tissues types such as flowers or roots.

**Extended Data Fig. 3:**
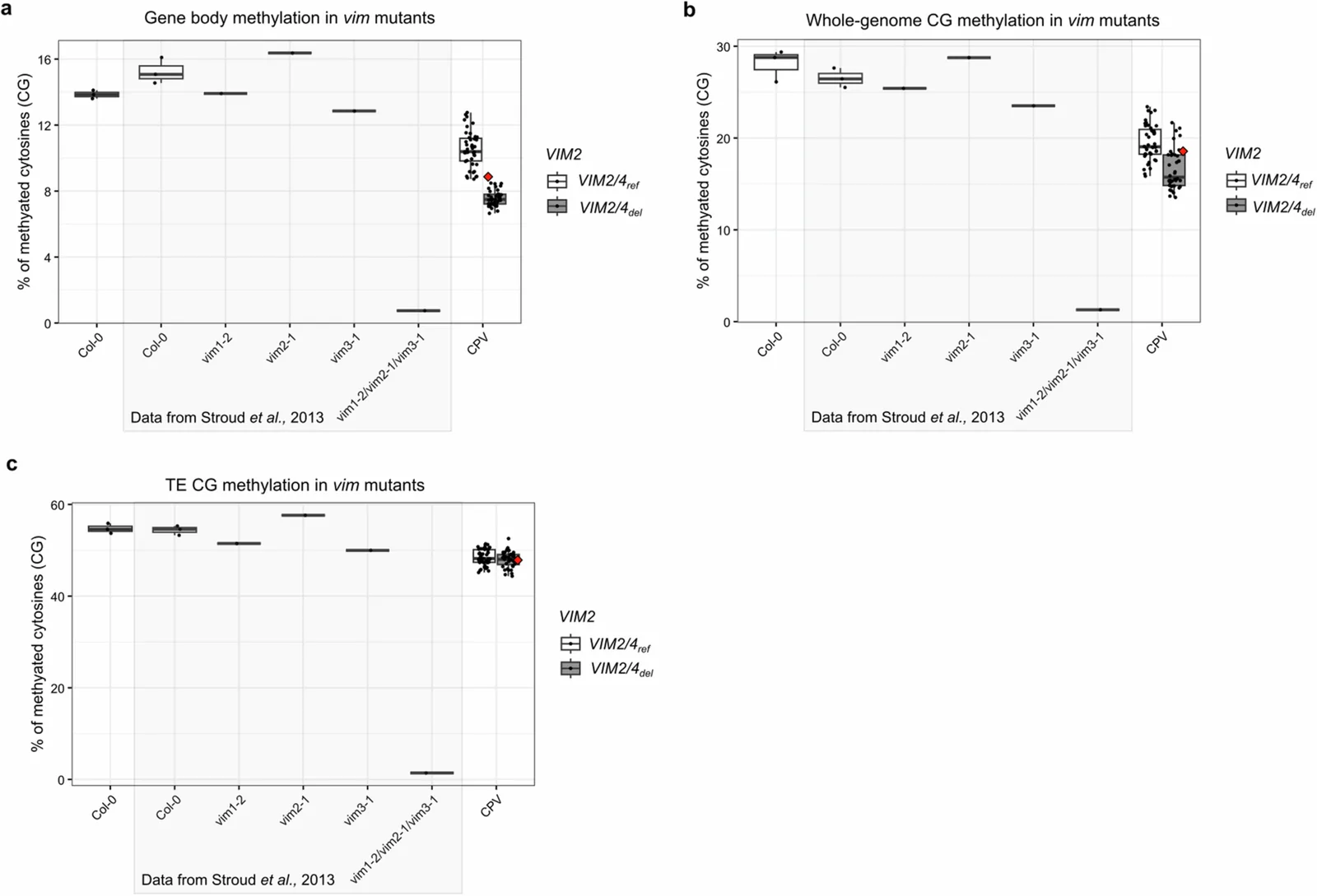
Re-analysis of DNA methylation data from Stroud et al., 2013. mCG methylation at gene body (gbM) (**a**), whole-genome (**b**), and all TEs (**c**) for the WGBS data from Stroud et al., 2013 (highlighted in a gray squared) and our WGBS data for Col-0 and the 83 CPV accessions segregating for the *VIM2/4del* allele. Only a single replicate per mutant was available and three replicates for the accession Col-0. Center lines of boxplots show the medians, box limits the 25th and 75th percentiles, whiskers extend 1.5 times the interquartile range from the 25th and 75th percentiles, dots represent individual data points. Red diamond represents normalized read count for Cvi-0. Number of individuals for each group is indicated below each box. P-value is indicated on the plot.

Although it has been shown that the triple *vim1/2/3* triple mutant leads to a strong reduction in mCG^53–55^, the mCG levels are higher in the *vim2* single mutant relative to wild type, while mCG levels are reduced in *vim1*, *vim3* and *vim1/2/3* (Extended Data Fig. 3a–c). This observation suggests that the function of VIM2 is more complex and that it could actually be a negative regulator of mCG, which would explain why overexpression of *VIM2/4* in the accessions with *VIM2/4del* is associated with gbM reduction. In addition, this hypothesis is consistent with results from Zhang and colleagues^56^ showing that the deletion is associated with increased stochastic mCG variation over generations.

**Extended Data Fig. 4:**
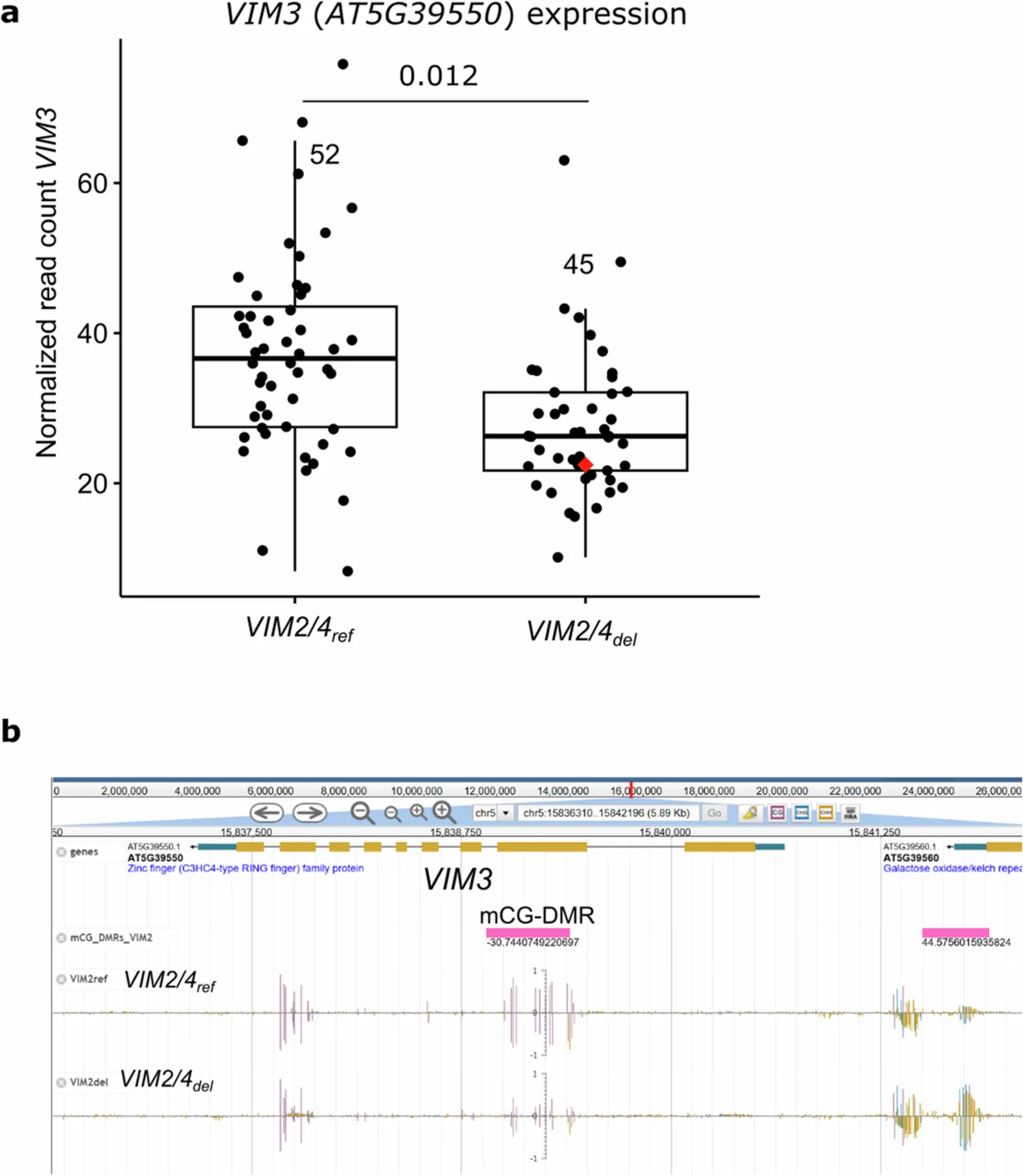
VIM3 expression and DNA methylation in VIM2/4ref and VIM2/4del background. **a**, Normalized read count for *VIM3* expression levels in 97 accessions from Santo Antão. The adjusted p-value from the RNA-seq analysis is displayed on the plot. Center lines of boxplots show the medians, box limits the 25th and 75th percentiles, whiskers extend 1.5 times the interquartile range from the 25th and 75th percentiles, dots represent individual data points. Red diamond represents normalized read count for Cvi-0. Number of individuals for each group is indicated below each box. P-value is indicated on the plot. **b**, Genome browser screenshot of the *VIM3* locus (AT5G39550, chr5:15,837,178.15,840,678) with the level of DNA methylation (red = CG, blue = CHG, yellow = CHH) and the DMRs identified between the *VIM2/4*_*ref*_ and *VIM2/4*_*del*_ genotypes.

GO term enrichment analysis on the 94 differentially expressed genes showed significant enrichment only for molecular functions related to methylcytosine binding (GO:0010429, GO:0008327 and GO:0010428), which was due to the three *VIM* homologues (*VIM2*, *VIM3* and *VIM4*) present in the differentially expressed gene list (>100-fold enrichment, Benjamini-corrected *P* value <3.8 × 10^−2^) (Extended Data Fig. 4a). Interestingly, a DMR overlaps the second exon of *VIM3*, with a lower CG methylation in VIM2/4_del_ genotype, which could potentially be responsible for the covariance in *VIM3* expression with the *VIM2*/*VIM4* deletion (Extended Data Fig. 4b). The fact that we do not see evidence of a change in gene expression in the categories for which we see a change in methylation is consistent with evidence that gene body methylation is not directly linked to gene expression^38^; it may instead moderate stochastic changes over longer timescales.

### TE DNA methylation in Cape Verde *Arabidopsis* is highly heritable

**Fig. 3:**
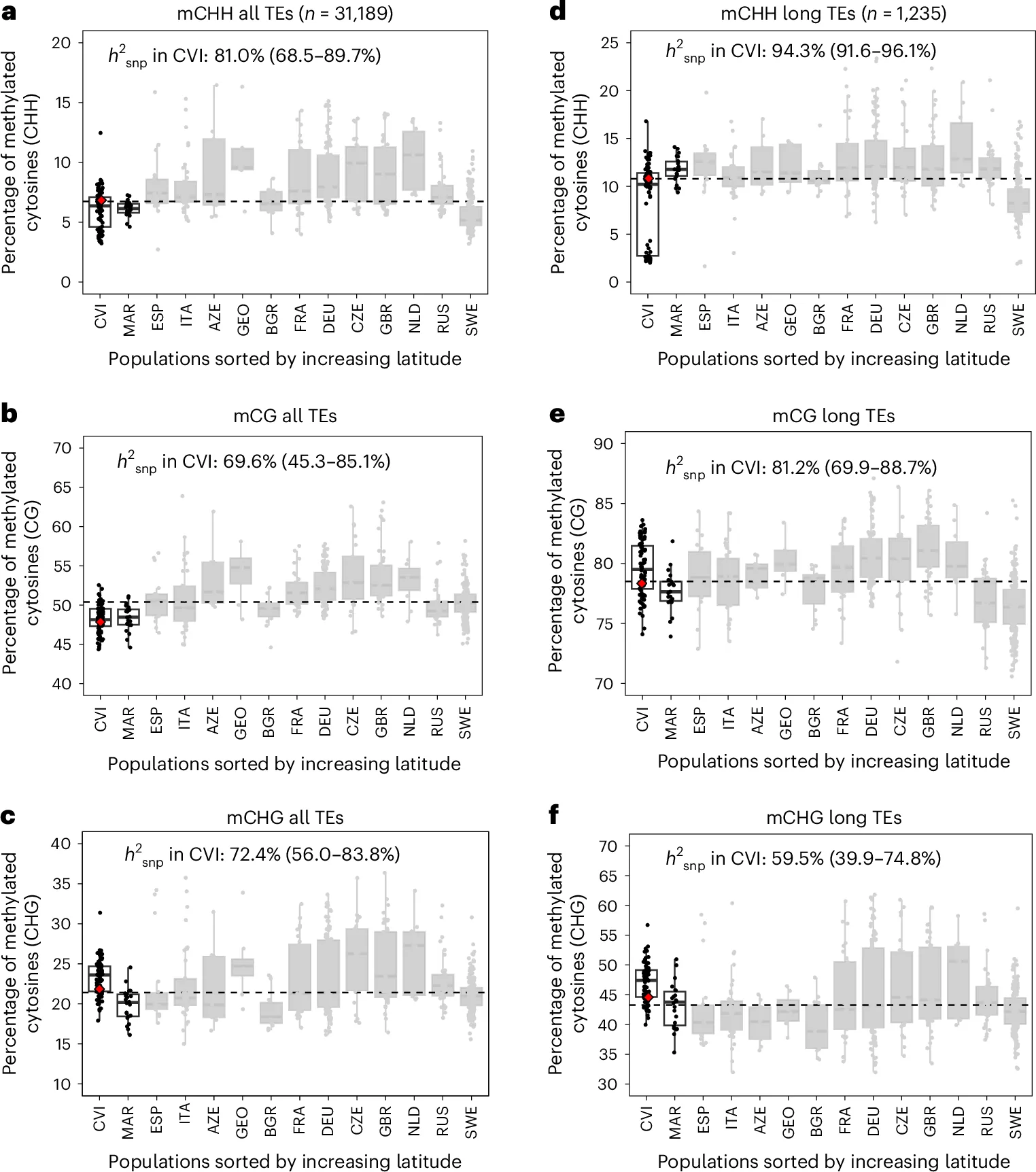
DNA methylation at TEs shows large heritable variance in CVI, indicating the presence of segregating genetic variation in the population. **a**–**f**, DNA methylation in CHH (**a**), CG (**b**) and CHG (**c**) contexts for all TEs and in CHH (**d**), CG (**e**) and CHG (**f**) for long TEs (>4 kb). Accessions are grouped by country (three-letter codes are the ISO names, except CVI; MAR indicates Morocco) and ordered by increasing latitude. The centre lines of box plots show the medians, boxes denote the 25th and 75th percentiles, whiskers extend 1.5 times the interquartile range from the 25th and 75th percentiles and dots represent individual data points. Dashed lines indicate the median value across all countries. The grey box plots represent the methylation levels from data (subset of 523 accessions of the 1001GP). The red diamond indicates the methylation levels of Cvi-0.

Next, we examined DNA methylation over TEs in CVI. We first assessed heritability of DNA methylation variation at all annotated TEs (31,189) and found SNP heritability to be 70–80% (Fig. 3a–c). We then considered separately long TEs (>4 kb), which are methylated specifically by the DDM1–CMT2 pathway^15^ so that methylation of these TEs may have a less complex genetic architecture. The 1,235 long TEs retained are mainly comprised of Class I long terminal repeat (LTR) Gypsy (42%) and Copia (17%) TEs, and of Class II DNA MuDR TEs (16%) (list in Supplementary Table 9). Long TEs are about twice as highly methylated as TEs overall and their methylation is more highly heritable in the CG and CHH contexts. Phenotypic variance was highest for mCHG and mCHH-methylated long TEs (Fig. 3d–f). SNP heritability was highest for mCHH (94.3%), followed by mCG (81.2%) in long TEs (Fig. 3c,e). For mCHG, the SNP heritability was higher when considering all TEs (72.4%) than long TEs (59.5%). Overall, the high heritability of TE methylation indicates genetic effects and suitability for mapping using GWAS. In subsequent analyses we focus on long TEs because methylation in these contexts is highly heritable.

### *CMT2* loss of function reduces mCHH over long TEs

**Fig. 4:**
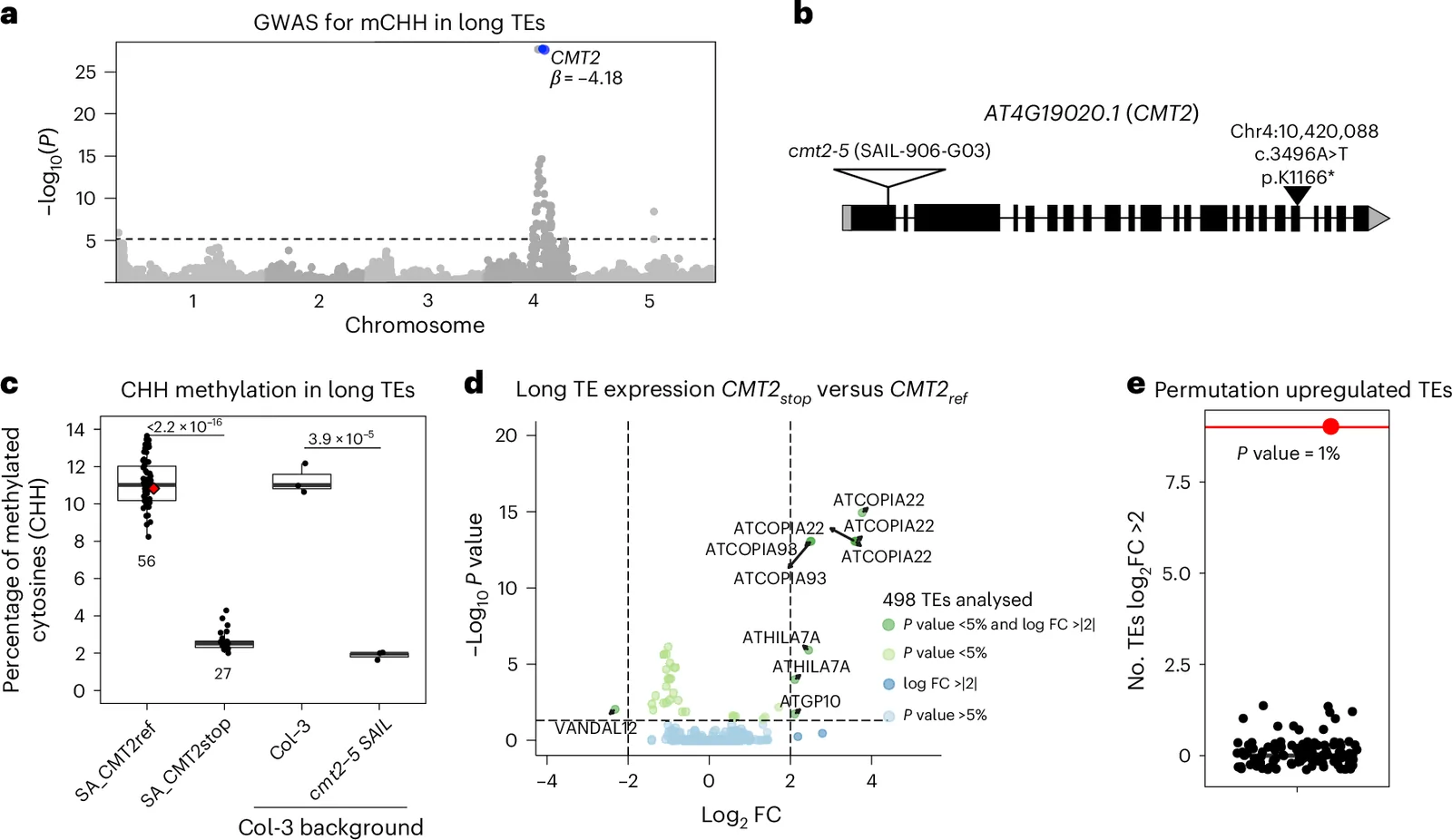
A nonsense variant in *CMT2* explains most of mCHH variation in long TEs. **a**, Manhattan plot of GWAS for mCHH in long TEs (>4 kb) shows a single major peak on chromosome 4. The nonsense SNP at *CMT2* (Chr4:10,420,088) is highlighted in blue and the Bonferroni threshold is indicated by a dashed line. **b**, Representation of the transcript model *AT4G19020.1* of *CMT2* includes exons (black boxes), UTRs (grey boxes), the location and Human Genome Variation Society (HGVS) nomenclature information of the nonsense variant that segregates in CVI (black triangle) and the T-DNA insertion of *cmt2-5* SAIL mutant (white triangle). **c**, Levels of mCHH in long TEs for the reference and *CMT2*_*stop*_ alleles in the Santo Antão (SA) natural population, and for the individuals with the T-DNA insertion line *cmt2-5* (SAIL_906_G03) and the corresponding Col-3 wild-type background accession. The red diamond represents mCHH level for Cvi-0. The number of individuals in each group is indicated below each box for Santo Antão accessions. Three biological replicates are represented for Col-3 and *cmt2-5*. We performed two-tailed *t*-tests to compare the means of the two groups (*P* values are indicated on the plot). The centre lines of box plots show the medians, box limits the 25th and 75th percentiles, whiskers extend 1.5 times the interquartile range from the 25th and 75th percentiles and dots represent individual data points. **d**, A volcano plot of long TE expression in accessions with the *CMT2*_*stop*_ versus *CMT2*_*ref*_ alleles. The names of the TEs with significant differential expression (<5% adjusted *P* value) and a log_2_fold change (FC) >|2| are displayed on the plot. Circles indicate TEs belonging to the same family. n.s., not significant. Five hundred and twenty-four TEs had at least ten reads across all accessions and could be used for differential expression analysis. **e**, The number of significantly upregulated TEs (log_2_FC >2) based on 100 permutations (black dots) with the observed values indicated in red.

GWAS using mCHH over long TEs as a phenotype revealed a clear peak on chromosome 4 (Fig. 4a), where the most significant SNP is a nonsense mutation in *CMT2* that introduces a stop codon and truncates the protein before a C-terminal methyltransferase domain (Chr4:10,420,088, A > T; Fig. 4b). Note that Cvi-0 has the *CMT2*_*ref*_ allele, indicating that the gene is functional in this accession (Fig. 4c). *CMT2*_*stop*_ is associated with a reduction of mCHH over long TEs of ≈82% (*β* coefficient of −4.18). *CMT2*_*stop*_ is present at 14 geographic sites with an overall frequency of 31% in Santo Antão (60 out 189 accessions) (Supplementary Fig. 5). Within those 14 sites, the allele is fixed in only one site with a very small sample size (*n* = 4), so that the frequency at this site may be poorly estimated. This pattern suggests that the allele may be subject to balancing selection due to a trade-off between its negative evolutionary effects through transposon mobilization and its potential positive effects due to increased heat tolerance of plants carrying the allele^44,57^. An additional peak in chromosome 5 contains two SNPs located in a TE (AT5TE52400) (Fig. 4a) that maps 472–749 kb downstream of *CMT2* on chromosome 4 in the Cape Verde genome assemblies. Therefore, the peak at chromosome 5 is an artefact of a discrepancy between the reference genome assembly (Col-0 TAIR10) used for mapping during SNP calling and the CVI genome organization. We did not find other significant peaks when *CMT2*_*stop*_ was used as a covariate (Supplementary Fig. 6). Comparison of CHH methylation in the *CMT2*_*stop*_ and *CMT2*_*ref*_ alleles in the natural population with a *cmt2-5* T-DNA insertion mutant, where mCHH was similar, provided further evidence that a truncation of CMT2 could explain the observed patterns. We also compared the difference in mCHH *CMT2* alleles in the natural population with a *cmt2-5* T-DNA insertion mutant and its wild-type background accession Col-0 and found the change in mCHH was similar, indicating that the natural *CMT2*_*stop*_ allele in CVI is most probably a null mutant (Fig. 4c). Overall, our data indicate that most of the variation in mCHH in CVI is driven by the natural *CMT2*_*stop*_ allele.

Next, we tested whether the difference in mCHH due to the *CMT2*_*stop*_ allele could affect long TE expression and found 38 significantly differentially expressed TEs, including 23 downregulated and 15 upregulated (Fig. 4d and Supplementary Table 10). The upregulated TEs are enriched in the LTR/Copia superfamily, with the families ATCOPIA22 and ATCOPIA93 showing the most significant upregulation (Fig. 4d). We tested whether this expression pattern was likely to be found by chance by conducting 100 permutations in which we attributed randomly chosen *CMT2* alleles within CVI subpopulation and found that the number of significantly differentially expressed TEs was always well above the permuted distribution (*P* = 0.01), supporting a significant association between the *CMT2*_*stop*_ variant and increased TE expression (Fig. 4e). To test whether the *CMT2*_*stop*_ variant was associated with more historical transposition events, we used mapping rate as a proxy for TE transposition and compared the percentage of whole-genome sequencing (WGS) reads mapping to TEs in all 190 accessions with and without CMT2_stop_. We found a significant increase in TE mapping in accessions with the *CMT2*_*stop*_ variant (Wilcoxon test statistic of 2,894, *P* value of 0.00432) (Supplementary Fig. 7). Overall, our results are consistent with higher TE expression and transposition in accessions carrying the *CMT2*_*stop*_ allele.

### *FBX5* downregulates mCG in long TEs

**Fig. 5:**
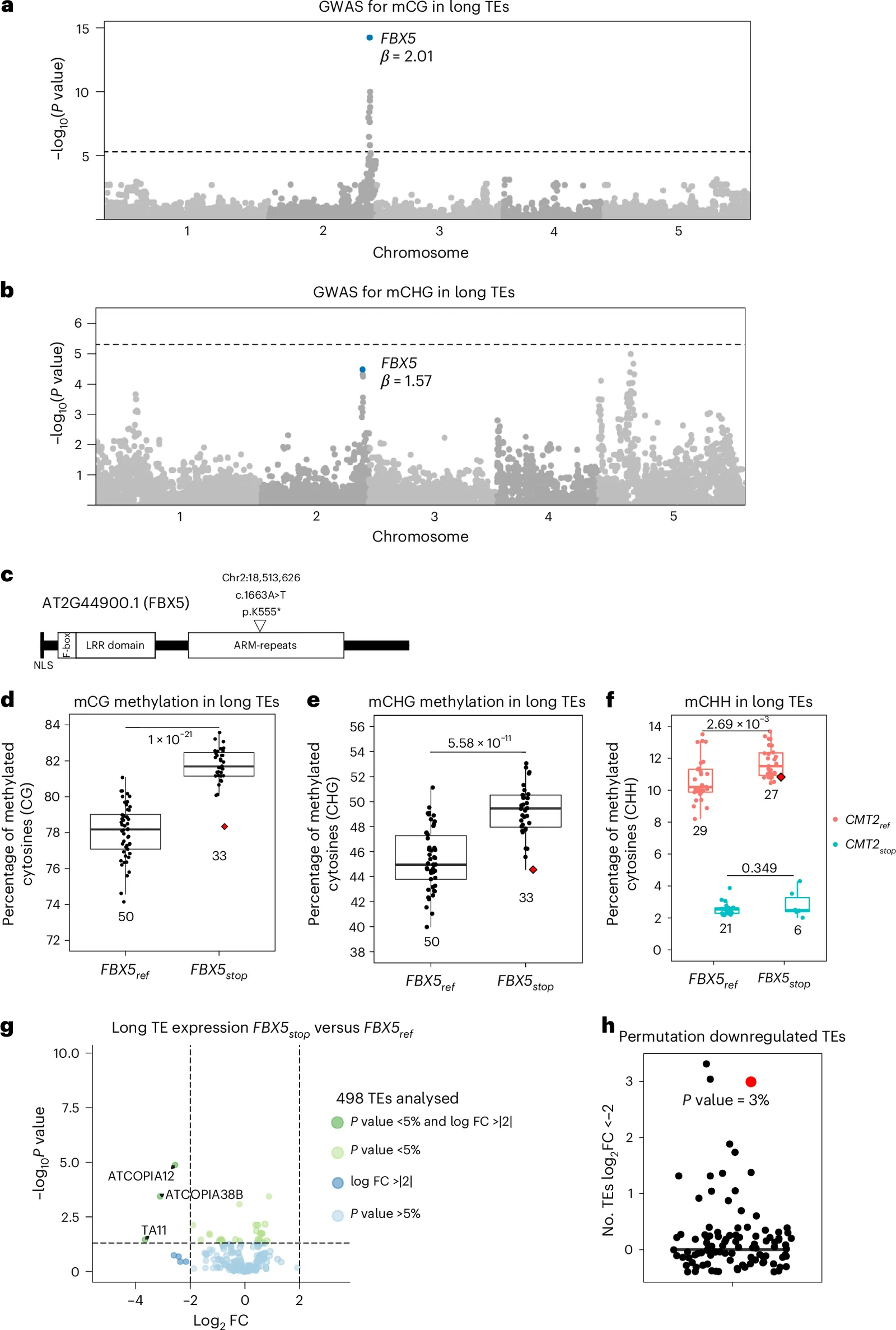
A premature truncation of *FBX5* is associated with decreased DNA methylation variation in CVI. **a**,**b**, Manhattan plots of the GWAS for mCG (**a**) and mCHG (**b**) on long TEs (>4 kb). Nonsense SNP at *FBX5* is highlighted in blue. The dashed line indicates the Bonferroni threshold. **c**, Representation of the protein domains of FBX5*.*The white triangle indicates the location of the nonsense mutation found in the natural CVI population with HGVS nomenclature information. The white boxes represent the protein domains (ARM, ARMADILLO; LRR, leucine-rich repeat; NLS, nuclear localization signal) as defined in ref. ^59^. **d**–**f**, The levels of mCG (**d**), mCHG (**e**) and mCHH (**f**) at long TEs for accessions with the reference (*FBX5*_*ref*_) and alternative (*FBX5*_*stop*_). In **f**, samples are separated based on their *CMT2* allelic state. The centre lines of box plots show the medians, box limits show the 25th and 75th percentiles, whiskers extend 1.5 times the interquartile range from the 25th and 75th percentiles and dots represent individual accessions. The number of individuals for each group is indicated below each box. The red diamonds represent the accession Cvi-0. Two-tailed Welch’s *t*-tests were performed to compare the means of the two groups in **d** and two-tailed Student’s *t*-tests were performed in **e** and **f**. *P* values are indicated in the plots. **g**, Volcano plot of long TE expression in accessions with the *FBX5*_*stop*_ versus *FBX5*_*ref*_ alleles. The names of the TEs with significant differential expression (<5% adjusted *P* value) and a log2FC >|2| are displayed on the plot. **h**, The number of significantly downregulated TEs (log_2_FC <−2) in permuted alleles within populations for 100 permutations (black dots) and the observed value indicated in red.

While the mean CHH methylation at long TEs was lower than for most other populations, mCG and mCHG levels at long TEs were higher in CVI (Fig. 3a–c). GWAS on mCG levels revealed one Bonferroni-significant peak at the end of chromosome 2 that includes a nonsense variant, which truncates the F-box Armadillo (Arm)-repeat gene *F-BOX PROTEIN 5* (*FBX5*) in the fourth exon (Chr2:18,513,626, A > T, p.K555*) (Fig. 5a,c). This gene is also known as *ARABIDILLO-1*. Here, we use *FBX5* since it is the gene name referenced in The Arabidopsis Information Resource (TAIR). *FBX5* is part of a E3 ubiquitin ligase complex that was shown to regulate lateral root development and abscisic acid-mediated inhibition of seed germination in *Arabidopsis*^58–61^. *FBX5* has not previously been implicated in DNA methylation.

**Extended Data Fig. 5:**
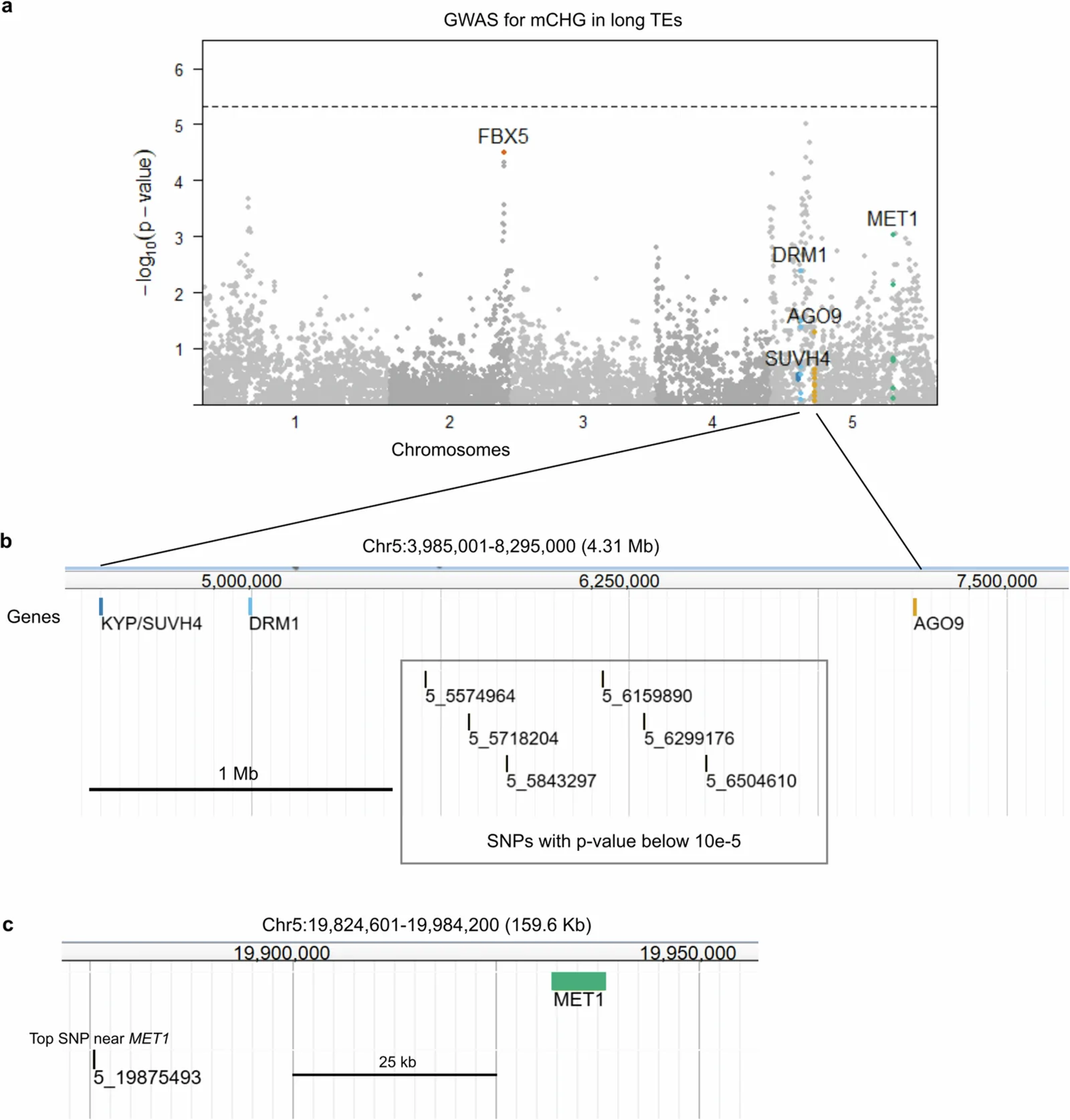
Candidate genes at chromosome 5 from the GWAS for mCHG at long TEs. **a**, Manhattan plots of the GWAS for mCHG in long TEs ( > 4 kb). SNPs surrounding (+/- 50 kb) *SUVH4, DRM1, AGO9*, and *MET1* are highlighted in dark blue, light blue, orange, and green, respectively. 10,318 SNPs were analyzed with a minor allele frequency (MAF) threshold of 5%. Bonferroni threshold is indicated by a dashed line. **b**, Zoom in on the chromosome 5 region containing *SUVH4*, *DRM1*, and *AGO9*. Top SNPs of chromosome 5 (below a p-value of 10e-5) are indicated with chromosome and position number. **c**, Most significant SNP (p-value = 9.5e-4) in *MET1* vicinity.

There were no Bonferroni-significant peaks for mCHG in long TEs. However, a peak at *FBX5* that includes the p.K555* variant was the one of the strongest signals (Fig. 5b). The strongest peak for mCHG at long TEs was a complex peak on chromosome 5 (Fig. 5b) within 500 kb of three genes known to be involved in DNA methylation: *AGO9*, *DRM1* and *SUVH4* (Extended Data Fig. 5a,b). A peak at *MET1* was also visible at the distal end of chromosome 5 (Extended Data Fig. 5a–c). Of the four candidate genes, we identified two containing coding variants with potential functions: a missense variant in *SUVH4* and two missense variants in *MET1* (Supplementary Table 11).

Accessions carrying *FBX5*_*stop*_ had 4.9% higher mCG (*β* = 2) and 6.3% higher mCHG levels (*β* = 1.57) (Fig. 5d,e). Although the *FBX5* region did not contain an obvious peak in the GWAS for mCHH (Fig. 4a and Supplementary Fig. 6), there was a moderate peak in the region. We examined the pattern within the CVI population and found evidence for a significant association of increased mCHH with *FBX5*_*stop*_ within the *CMT2*_*ref*_ background (Fig. 5f), suggesting that *CMT2* is epistatic to *FBX5* in the regulation of mCHH. Although the effect of *FBX5* is clearest in the CG context, trends in our data and another recent publication^56^, where peaks for mCHH and mCHG were identified in the same region in the Cvi-0 x Ler-0 RIL population, suggest that *FBX5* probably exerts a more general effect on DNA methylation. Considering the fact that F-box proteins regulate at the post-translational stage, we did not expect transcriptional regulation of MET1 by FBX5. However, to test the hypothesis of a transcriptional effect of FBX5 on MET1, we looked at differentially expressed genes between lines segregating for FBX5 nonsense variant and did not find any significant changes in expression.

*FBX5*_*stop*_ is present in 13 populations with an overall frequency of 33% (62 out 189 accessions) in Santo Antão (Supplementary Fig. 8). We did not find other significant peaks when *FBX5*_*stop*_ was used as a covariate (Supplementary Fig. 9). Surprisingly, although Cvi-0 is homozygous for the *FBX5*_*ref*_ allele, it is at the extreme low end of the range of mCG and mCHG (Fig. 5d,e). This suggests that novel mutations may have arisen during the many successive propagations in artificial conditions since its initial collection 40 years ago in CVI and that some aspects of the Cvi-0 epigenome may not be representative of the natural population. To assess whether high impact variants in *FBX5* occur outside of CVI, we used the polymorph1001 webtool (ref. ^62^) and identified four accessions in addition to Cvi-0 with high impact mutations. One Russian accession (seqID 18696) contains a nonsense mutation (Chr2:18,513,091 C > A) and three accessions from the UK (seqIDs 4807, 7111 and 7342) contain a splice site mutation within the 3′UTR (Chr2:18,516,122 A > G). These data suggest that high impact polymorphisms at *FBX5* are present outside of CVI but occur at low frequency.

Converse to the pattern for *CMT2*_*stop*_, the *FBX5*_*stop*_ allele was associated with increased DNA methylation over long TEs, in line with a de-repressive function of *FBX5* reflected in the increased mCG and mCHG levels in the mutant (Fig. 5g). We found 30 significantly differentially expressed TEs between the two allelic states in the CVI population, including 16 downregulated and 14 upregulated TEs (Fig. 5g and Supplementary Table 12). The downregulated TEs belong to several superfamilies, including DNA/MuDR and LTR/Copia elements (Supplementary Table 12). AT2TE22635 (TA11 family, Line/L1 superfamily) showed the strongest downregulation in the *FBX5*_*stop*_ background (Fig. 5g). Significantly upregulated TEs were all from the LTR/Gypsy superfamily, with ten ATHILA and four ATLANTYS3 family TEs (Supplementary Table 12). We tested whether this pattern was likely to be found by chance using a permutation analysis wherein we randomly attributed *FBX5* alleles within resampled individuals. We found that the number of downregulated TEs is greater than that ever found in 100 permutations (*P* = 0.01), supporting that the *FBX5* variant is associated with significant downregulation of TE expression (Fig. 5h). We also tested the potential difference in TE transposition events by using it as a proxy for the percentage of WGS reads mapping to TEs in *FBX5*_*stop*_ versus *FBX5*_*ref*_. While the mean percentage of reads mapping to *FBX5*_*stop*_ was reduced as would be expected from its increased DNA methylation, the trend was not significant (Wilcoxon test statistic of 4142, *P* value of 0.625), suggesting that a mild change in mCG is not sufficient to produce a detectable difference in transposition rates in our sample (Supplementary Fig. 10).

**Fig. 6:**
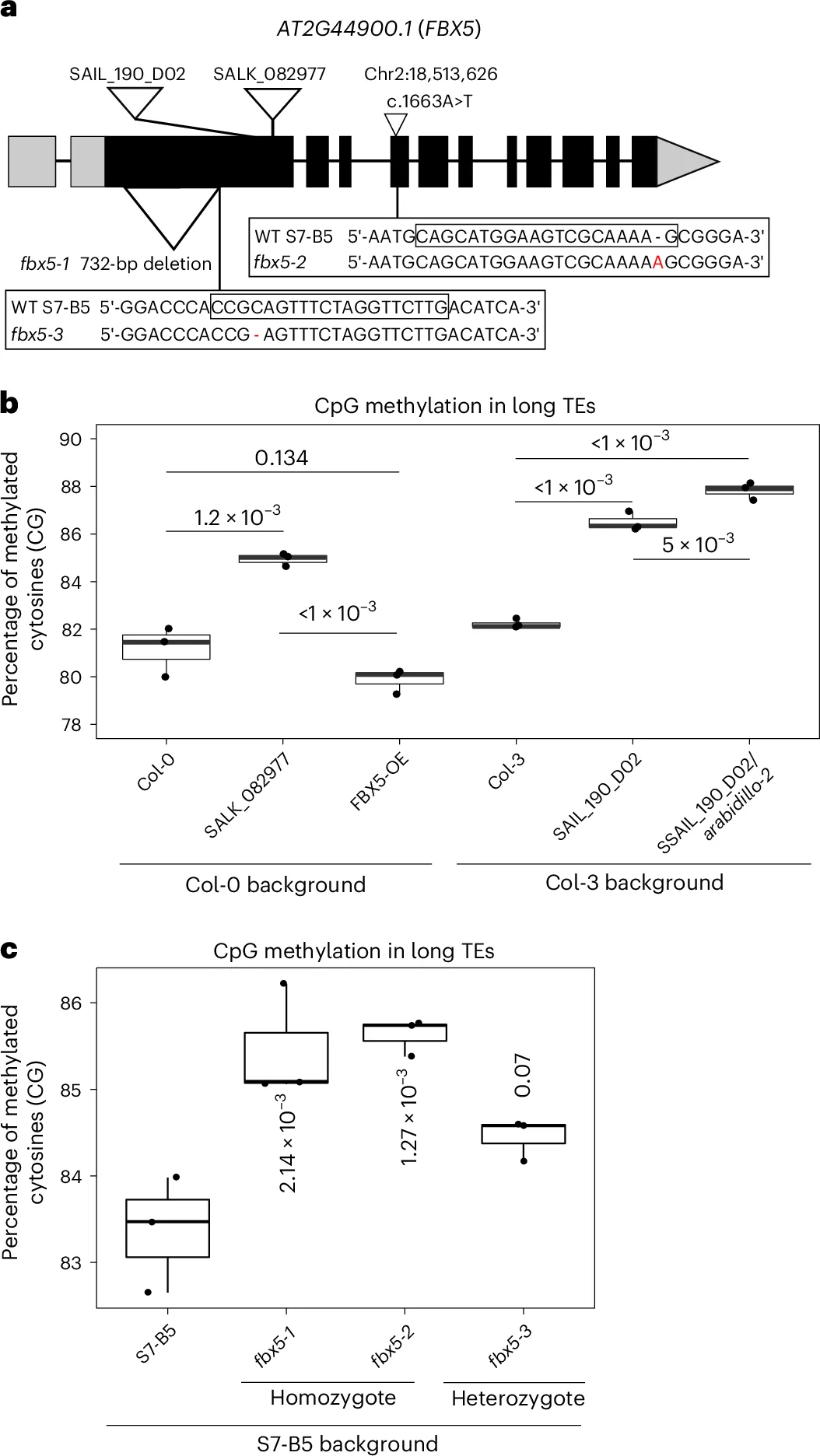
*FBX5* is a negative regulator of DNA methylation of long TEs. **a**, Representation of the transcript model *AT2G44900.1*. The small white triangle indicates the location of the truncation mutation found in the natural population with HGVS nomenclature information. The grey boxes represent UTRs and black boxes the exons. T-DNA insertion lines are represented by white triangles with their stock names. The genotype of the three CRISPR–Cas-9 based frameshift mutants (*fbx5-1*, *fbx5-2* and *fbx5-3*) are indicated in comparison to the background CVI accession used (S7-B5). The line *fbx5-1* contains a deletion of 720 bp (white triangle). The box in the wild-type (WT) S7-B5 sequence indicates the target site for CRISPR–Cas9, and red characters in mutants show the indel mutations. **b**, mCG of long TEs increases in *FBX5* T-DNA insertion lines SALK_082977, SAIL_190_D02 and SAIL_190_D02/arabidillo-2 (SAIL_162_B11) and decreases in the overexpression line (FBX5-OE), relative to the accessions used for transformation (Col-0 for SALK and Col-3 for SAIL). For each background, an ANOVA was performed followed by a Tukey’s test (two tailed). *P* values are indicated on the plot. **c**, The level of mCG of long TEs is increased in three independent *fbx5* CRISPR–Cas9 lines relative to S7-B5 (*FBX5*_*ref*_) (three biological replicates per line). The line *fbx5-3* is heterozygous for the mutation and shows an intermediate phenotype. An ANOVA was performed followed by a Dunnett’s test (two tailed) with S7-B5 used as control. P values are indicated in the plot. The centre lines of box plots show the medians, box limits show the 25th and 75th percentiles, whiskers extend 1.5 times the interquartile range from the 25th and 75th percentiles and dots represent individual accessions.

**Extended Data Fig. 6:**
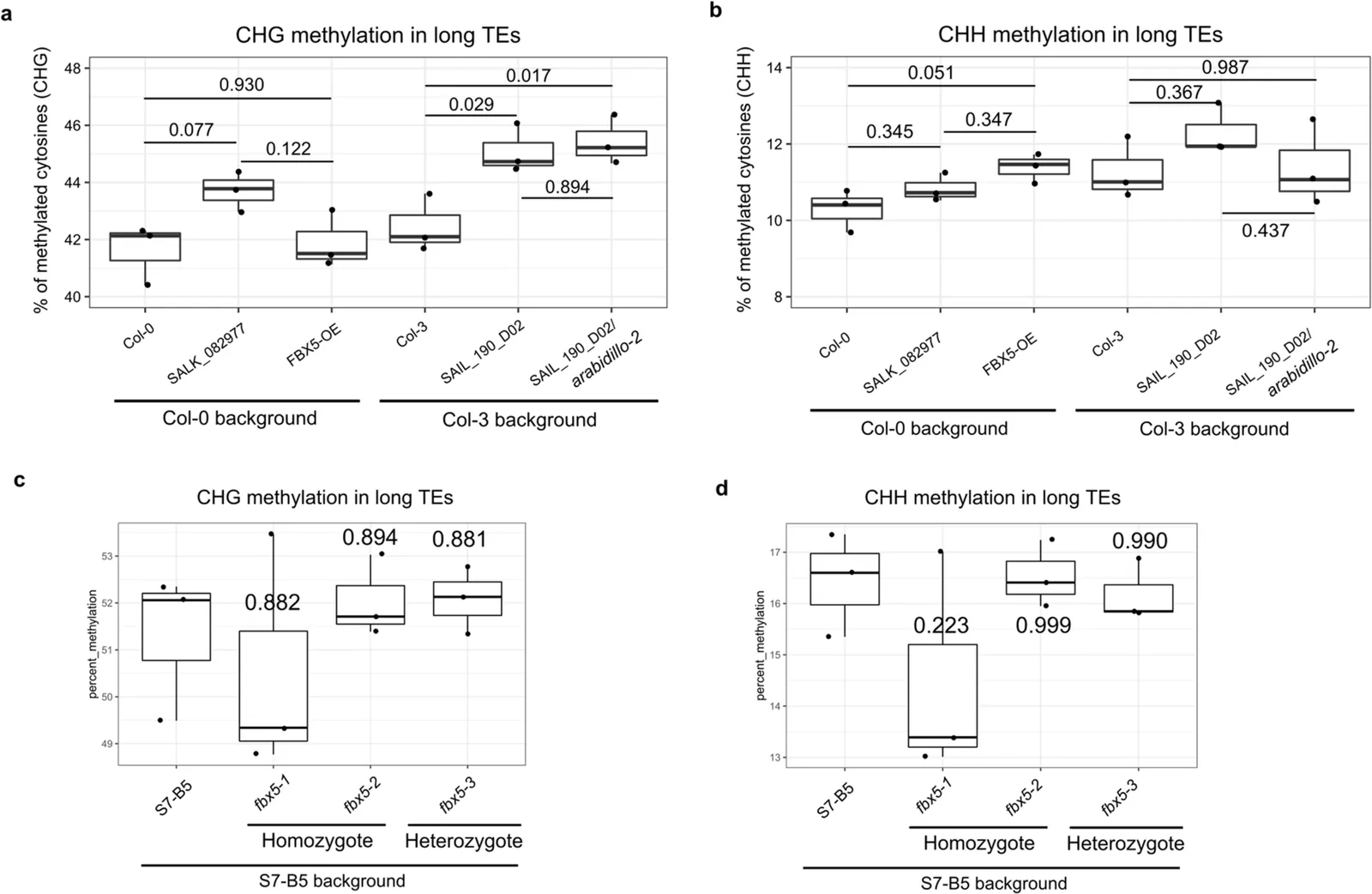
DNA methylation levels in FBX5 overexpressing and mutant Arabidopsis. Levels of mCHG (**a**) and mCHH (**b**) in long TEs for *fbx5* T-DNA insertion lines SALK_082977, SAIL_190_D02, SAIL_190_D02/arabidillo-2 (SAIL_162_B11), overexpression line (FBX5-OE), and the WT background accessions used for transformation (Col-0 for SALK and Col-3 for SAIL. For each background, an ANOVA was performed followed by a Tukey’s test (two-tailed). P-values are indicated on the plot. Levels of mCHG (**c**) and mCHH (**d**) in in long TEs for three independent *fbx5* CRISPR lines in the S7-B5 background (three biological replicates per line). An ANOVA was performed followed by a Dunnett’s test (two-tailed) with S7-B5 used as control (p-values are indicated on the plot). Note that the y-axis labelling does not start at zero. Center lines of boxplots show the medians, box limits the 25^th^ and 75^th^ percentiles, whiskers extend 1.5 times the interquartile range from the 25^th^ and 75^th^ percentiles, dots represent individual data points.

To confirm the role of *FBX5* in DNA methylation, we performed whole-genome bisulfite sequencing in two independent *fbx5* T-DNA insertion lines (SAIL_190_D02 and SALK_082977), a double mutant of *fbx5* and its close homologue *arabidillo*-2 (SAIL_190_D02/SAIL_162_B11) and a transgenic *FBX5* overexpression line (FBX5-OE)^58^ (Fig. 6a). We found increased methylation levels for mCG (Fig. 6b) and mCHG (Extended Data Fig. 6a) in the T-DNA insertion lines. Consistent with this general pattern, mCG was lower, although not significantly reduced, in the *FBX5* overexpression line (Fig. 6b). The homologue *ARABIDILLO-2* also appears to impact mCG based on the higher mCG level in the double mutant compared with the single mutant (Fig. 6b). We also generated CRISPR *fbx5* knock-out lines in a CVI genetic background using the CVI accession S7-B5 that contained *CMT2*_*ref*_ and *FBX5*_*ref*_ (Fig. 6a). We produced two lines that were homozygous for the Cas9-induced mutations (*fbx5-1* and *fbx5-2*) and one heterozygous line (*fbx5-3*). Homozygous lines show an increase in long TE mCG, while the heterozygous line shows an intermediate phenotype, indicating haplo-insufficiency (Fig. 6c). No significant methylation changes were observed overall for CHG and CHH contexts, indicating that any impact in these contexts may change more subtly and stochastically over generations (Extended Data Fig. 6c,d). These results confirm that *FBX5* is a negative regulator of DNA methylation, most strongly in the CG context.

### The DNA methylation variants arose recently and spread rapidly

**Fig. 7:**
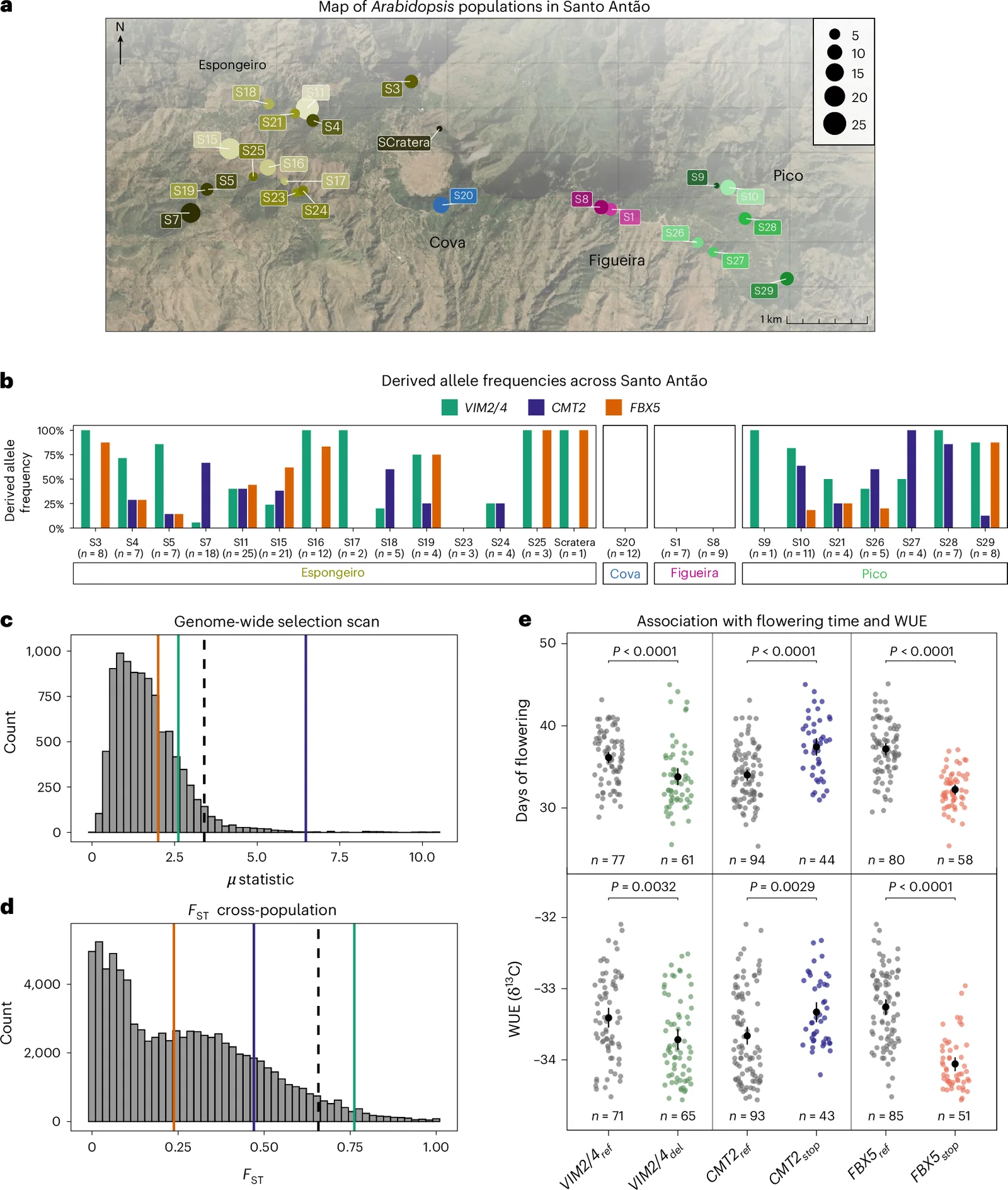
Signatures of selection and phenotypic associations with each of the methylation variants. **a**, A map of Santo Antão showing the geographic distribution of 24 sampling sites within the island population. Accessions are clustered into four main subpopulations (Espongeiro, Cova, Figueira and Pico), with point size proportional to sample size. **b**, Derived allele frequencies for *CMT2*, *FBX5* and *VIM2/4* across the 24 sampling sites, arranged according to the four major subpopulations. Total sample sizes (*n*) for each site are indicated below each site name. **c**,**d**, Genome-wide scans for positive selection using the RAiSD *μ* statistic (**c**) and population differentiation using pairwise *F*_ST_ between segregating (Pico/Espongeiro) and ancestral-fixed (Cova/Figueira) subpopulations (**d**). The solid coloured vertical lines highlight the three methylation variants, while dashed black lines indicate the 95th percentile significance thresholds. **e**, The impact of allelic variation on fitness-related traits. The presence of the derived alleles (*VIM2/4*_*deletion*_, *CMT2*_*stop*_ and *FBX5*_*stop*_) is significantly associated with shifts in flowering time (days to flowering; top) and WUE (δ^13^C; bottom) compared with the ancestral reference (ref) alleles. The black dots and error bars denote the mean and 95% CI, respectively. Significance (*P* values) was determined using linear models, with the *FRI*_K232X_ incorporated as a covariate for flowering time. Sample sizes (*n*) for each allelic class are indicated at the bottom. Basemap generated with Esri World Imagery.

We examined the geographic distributions and inferred the ages of the methylation-impacting variants across Santo Antão (Fig. 7a). The derived variants *VIM2/4*_*del*_, *CMT2*_*stop*_ and *FBX5*_*stop*_ are absent from the Cova and Figueira subpopulations, which we previously showed best represent the original colonizers^48,63,64^. Within the more drought-prone Pico and Espongeiro regions where *A. thaliana* populations expanded more recently, the variants are at intermediate frequencies (Fig. 7b). While *VIM2/4*_*del*_ is at the highest frequency overall (46%) and is fixed in several individual stands, *CMT2*_*stop*_ and *FBX5*_*stop*_ are at lower frequencies (*CMT2*_*stop*_, 38%*; FBX5*_*stop*_, 33%), widespread across stands, but only rarely fixed in individual stands (Fig. 7b and Supplementary Figs. 2, 5 and 8). We used coalescent reconstructions to estimate ages for the three variants, which were all much younger than the estimated colonization at approximately 5–7 kya (refs. ^65,66^). We estimated *VIM2/4*_*del*_ emerged between 553 (95% confidence interval (CI) 264–909) years ago and 1,033 (95% CI 599–1,651) years ago and increased in frequency with a selection coefficient of *s* = 9.2 × 10^−3^ (Supplementary Fig. 11a). We estimated *CMT2*_*stop*_ arose between 490 (95% CI 280–766) years ago and 2,207 (95% CI 1,428–2,822) years ago with a selection coefficient of *s* = 6.3 × 10^−3^ (Supplementary Fig. 11b). Similarly, we inferred *FBX5*_*stop*_ arose between 463 (95% CI 223–894) years ago and 1,786 (95% CI 881–2613) years ago with *s* = 1.4 × 10^−2^ (Supplementary Fig. 11c). Next, we assessed whether the methylation variants displayed evidence for selective sweep signatures based on extreme allelic differentiation and linkage disequilibirium-based sweep signatures. We calculated two summary statistics across the genome: pairwise *F*_ST_ and RAiSD *μ*statistic, and compared the values for the methylation variants with the genome-wide distributions of the statistics. *CMT2*_*stop*_ was a significant outlier in this test (RAiSD *μ* statistic of 6.47, empirical *P* value of 3.5 × 10^−3^) (Fig. 7c). *VIM2/4*_*del*_ was in the extreme tail of the distribution of allelic differentiation (*F*_ST_ = 0.75, empirical *P* value of 2.0 × 10^−2^) (Fig. 7d). Overall, selection coefficients inferred from historical reconstruction and results from selection scans suggest the methylation variations spread rapidly after they arose, consistent with positive selection.

### Methylation variants are associated with alternative drought adaptation strategies

Plants can adapt to drought-prone environments through multiple strategies, including drought resistance in which they close their stomata to maximize water use efficiency (WUE) and slow growth to persist through the drought; or by drought escape, in which they maximize gas exchange and rapidly progress to flowering^67,68^. We investigated potential associations between the methylation-affecting variants we identified and traits related to drought resistance and escape^48,63^. We found that all three variants were significantly associated with flowering time and WUE (Fig. 7e). While *CMT2*_*stop*_ was associated with a drought resistance strategy, characterized by higher WUE (*β* = 0.33, SE=0.11, *p* = 0.003) and slower flowering time (β = 2.91, s.e.m. 0.64, *P* < 0.001), *VIM2/4*_*del*_ and *FBX5*_*stop*_ were associated with a drought escape strategy, exhibiting significantly lower WUE (*VIM2/4*_*del*_: *β* = −0.31, s.e.m. 0.10, *P* = 0.003; *FBX5*_*stop*_: *β* = −0.80, s.e.m. 0.08, *P* < 0.001) and earlier flowering (*VIM2/4*_*del*_: *β* = −2.97, s.e.m. 0.58, *P* < 0.001; *FBX5*_*stop*_: *β* = −4.66, s.e.m. 0.49, *P* < 0.001). The results for *CMT2* are consistent with previously proposed roles of *CMT2* in varying climates^44,45^. Together, these results suggest that positive selection on genes involved in distinct DNA methylation pathways may facilitate alternative ecological strategies to cope with the local climate in Santo Antão.

## Discussion

Mutations that impact DNA methylation machinery have the potential to produce rapid genome-wide changes due to their widespread genomic impacts^69,70^. Our GWAS in CVI revealed high impact genetic polymorphisms for two known players of DNA methylation, *VIM2/4* and *CMT2*, and a novel player, *FBX5*.

A 2.7-kb deletion between the two *VARIANT IN METHYLATION* homologues *VIM2* and *VIM4* reduced gbM in CVI. In *Arabidopsis*, there are five *VIM* homologues (*VIM1–5*)^52,71^, which regulate DNA methylation by recruiting MET1 to hemi-methylated CG sites for maintenance methylation. This is accomplished with a PHD domain, which enables reading of core histones and an SRA domain, which facilitates binding to methylated DNA at histone H3 (reviewed in ref. ^72^). The triple mutant *vim1/2/3* phenocopies the *met1* null allele mutant through complete loss of mCG^54,55^. Whereas a family of *VIMs* encode proteins required for methylation regulation in plants, in mammals a single orthologue, *UHRF1*, encodes a protein responsible for these functions^73,74^. Expansion of this gene family in plants probably facilitated separation of functions and higher specificity. In the CVI population, we found differential gene body methylation between *VIM2/4* alleles was significantly enriched for Cul4–RING E3 ubiquitin ligase complexes, which include genes involved in environmental response traits, including light signalling, abscisic acid signalling, flowering, metabolism and DNA repair. The mammalian homologue of the VIMs, *UHRF1*, is involved in DNA repair^75^, suggesting that VIMs could also have such functions in plants. We also found a gbM GWAS signal near VIM3, but this appears to be an artefact of the high sequence homology of the *VIM4* and *VIM3* promoter regions, supporting a model where *VIM3* arose recently through an interchromosomal duplication of *VIM2* or *VIM4*. Our results for the *VIM2/4* region point to additional hypotheses that could be tested related to the role of gene body methylation in stress response evolution.

We also identified genetic variants that impacted methylation of TEs. mCHH levels on TEs, especially long TEs, had a bimodal distribution, due to an allele that disrupts *CMT2* segregating in CVI. Other *CMT2* null alleles have been identified worldwide and their distribution is correlated with climate, suggesting that *CMT2* natural variation could be adaptive^44,76,77^. Further, two studies have shown that *cmt2* mutants are more resistant to heat stress and one study demonstrated a higher resistance to UV-B radiation^44,57^. However, disruption of CMT2 is also expected to reduce silencing of TEs, resulting in a potential increase in genome instability. A previous study found that COPIA transposon expression increased following heat stress in Col-0-*cmt2* and in the Kyoto accession, which carries a natural *CMT2* mutation^78,79^. Since CMT2 is involved in maintenance methylation, its disruption probably does not immediately remove methylation marks; instead release of COPIA elements may be aided by heat exposure and other stresses over generational time^80,81^. Consistent with this, we found that several COPIA elements were expressed in the *CMT2* null background in the CVI natural population, which is exposed to heat and drought stress. The *CMT2*_*stop*_ allele is segregating at intermediate frequencies across nearly all CVI subpopulations we sampled (Fig. 7a,b), suggesting they may be evolving under balancing selection where the allele provides a positive impact on resistance to heat and UV stress, but a negative impact on genome stability. An alternative possibility derives from the argument that the increased mutation rate due to TEs may be beneficial in a population adapting to a novel, harsh environment^80,82,83^. Under this logic, the increased mutation rate itself may have been beneficial in the variation-limited CVI population.

We discovered a novel player in DNA methylation, *FBX5* (*ARABIDILLO-1*), that impacts CG methylation over TEs. Similar to the *CMT2*_*stop*_ variant in CVI, the *FBX5*_*stop*_ allele is segregating in almost all subpopulations. The mild increase of mCG in accessions with the *FBX5* null allele was associated with the downregulation of several TEs, including COPIA elements, indicating that even a mild increase in DNA methylation can potentially lead to changes in TE repression. *FBX5* and its close homologue *ARABIDILLO-2* were previously shown to regulate lateral root development and abscisic acid-mediated inhibition of seed germination^58–61^. This indicates a pleiotropic effect of *FBX5* and *ARABIDILLO-2* on plant molecular and organismal phenotypes with potential adaptive consequences. Interestingly, the mammalian orthologue of FBX5, β-catenin, interacts physically with the mammalian orthologue of MET1, DNMT1, assuring mutual stabilization of the two proteins^84^. Considering that protein sequence similarity between FBX5 and β-catenin is high^85^, disruption of FBX5 as found in CVI could potentially affect MET1 activity. Further investigation is needed to determine how *FBX5* regulates DNA methylation.

Plants are known to use multiple alternative strategies to adapt to drought, including drought avoidance and drought resistance^67,68^. Methylation variants we identified appear to have spread rapidly in CVI and were associated with different drought adaptation strategies: *CMT2*_*stop*_ was associated with drought-avoidance traits (higher WUE and later flowering), while *FBX5*_*stop*_ and VIM2/4_del_ were associated with drought-escape traits (lower WUE and faster flowering). Although *CMT2*, *VIM2/4* and *FBX5* all influence DNA methylation, they affect distinct components of the epigenetic machinery rather than constituting a single linear pathway. CMT2 primarily regulates CHH methylation over heterochromatic TEs, VIM2/4 acts in CG maintenance over gene bodies and FBX5 appears to influence CG methylation over TEs through a potentially indirect mechanism. Overall, this suggests the loci underlie parallel evolutionary routes that converge on epigenetic remodelling. Thus, these loci probably represent largely independent paths towards mechanistically distinct perturbations of DNA methylation.

The bottleneck that occurred during colonization of CVI eliminated nearly all segregating variation; as a result, these functional variants arose de novo within the islands. In evolutionary genetics, it is often expected that phenotypic variance should be low in populations with low genetic diversity. However, this case exemplifies how phenotypic variance can be as high or higher in a population with low genetic diversity—where higher resolution mapping may be possible—compared with populations with much higher genetic diversity (Figs. 1b and 3). Mutations that shape DNA methylation variation in CVI have considerable effects on methylation levels and gene function, disrupting DNA methylation regulators (CMT2 K1166* and FBX5 K555*) or removing a large shared regulatory region between *VIM2* and *VIM4*. However, the genes involved tend to impact DNA methylation in indirect ways. CMT2 functions in maintenance of DNA methylation so that its absence may act to quantitatively reduce rather than eliminate DNA methylation over generations. FBX5 probably interacts with MET1 based on studies of the human homologue, β-catenin^84^. We did not observe DNA methylation phenotypes associated with major effect mutations in key DNA methylation genes such as *DDM1* or *MET1*. This suggests natural variation may be more readily maintained in nature when it produces subtle effects on DNA methylation, allowing for tuning of DNA methylation, which may have potential adaptive effects at some loci, rather than the large-scale loss that would result from disruption of key regulators of methylation such as *MET1* or *DDM1*. Overall, our findings point to several potential areas for further investigation to dissect the roles of DNA methylation in environmental response.

## Methods

### Growth conditions

Plants were grown on soil (Brill Classic, Brill Substrate GmbH) and stratified for 1 week at 4 °C. Pots were transferred in a Bronson chamber (Bronson Climate BV) with 12 h day/12 h night photoperiod, light intensity 200 µmol m^−^^2^ s^−1^, temperature 21 °C day /14 °C night.

### Plant material

Seeds of accessions from Cape Verde and Morocco are available in our seed stock (Max Planck Institute for Plant Breeding Research, Cologne, Germany)^48,86^. Seeds of *fbx5* T-DNA mutants from Syngenta Arabidopsis Insertion Library (SAIL_190_D02) and SALK (SALK_082977), *arabidillo-2* (SAIL_162_B11), double mutant *fbx5/arabidillo-2* (SAIL_190_D02/SAIL_162_B11) and *FBX5* overexpressing lines were provided by Juliet Coates^58^. Seeds from the 1001GP accessions Dör-10 and UKID116, the *cmt2* T-DNA mutant (SAIL_906_G03) and Col-0 (CS76114) were ordered from the Nottingham Arabidopsis Stock Center. T-DNA insertions were genotyped by PCR (primers listed in Supplementary Table 13).

### DNA sequencing

Raw sequencing read data from WGS of the 190 Cape Verde (Santo Antão) accessions originate from published work^48^ and are available on European Nucleotide Archive under accession code PRJEB39079 (Supplementary Table 14).

### GWAS analysis

SNP calling was performed using the MPI-SHORE pipeline^87^ with minor modifications. The detailed script is available at via GitHub^88^.

VCF processing and GWAS analysis was performed as detailed in the GitHub repository^89^. In brief, non-biallelic, singletons and indel variants were filtered out of the VCF file before analysis. Only SNP calls with a read coverage DP ≥3 and genotype quality GQ ≥25 were considered. To characterize the genetic architecture of the different traits, we used a Bayesian sparse LMM in GEMMA (v0.94)^50^. We ran Markov chain Monte Carlo (MCMC) with 10,000,000 sampling steps and 2,500,000 burn-in iterations. We calculated the median and 95% CI for the proportion of variance in phenotypes explained by available genotypes across ten runs. A minor allele frequency (MAF) of 5% was applied so that the final set 10,321 analysed SNPs were used for GWAS. A univariate LMM was used for association testing with the software GEMMA^90^ according to the model

$${\bf{y}}={\bf{W}}{\bf{\alpha}} +{\bf{x}}\beta +{\bf{u}}+\,{\bf{\epsilon}} ;{\bf{u}} \sim \,{MV}{N}_{n}(0,{\lambda \tau }^{-1}K),\,{\bf{\epsilon}} \sim {MVN}(0,{\tau }^{-1},{I}_{n})$$

where **y** is an *n*-vector of quantitative traits (weighted DNA methylation level) for *n* individuals; **W** = (**w**_1_*; · · ·,* **w**_*c*_) is an *n* *×* *c* matrix of covariates (fixed effects) including a column of 1 s; ***α*** is a *c*-vector of the corresponding coefficients including the intercept; **x** is an *n*-vector of marker genotypes; *β* is the effect size of the marker; **u** is an *n*-vector of random effects; ϵ is an *n*-vector of errors; *τ−*1 is the variance of the residual errors; *λ* is the ratio between the two variance components; *K* is a known *n* *×* *n* relatedness matrix and *I*_*n*_ is an *n* *×* *n* identity matrix. MVN*n* denotes the *n*-dimensional multivariate normal distribution. We assessed SNP significance using the Bonferroni threshold (−log_10_(0.05/number of SNPs tested)). The standardized kinship matrix was used.

To integrate the deletion status at *VIM2/4* in the VCF file, which contains only SNPs, an artificial SNP coding for the absence/presence of the deletion was added manually at the approximate position of the deletion.

The *β* coefficients for the different SNPs of interest were obtained from the LMM of the GWAS and provided the effect size of each SNP on the phenotype. The *β* coefficients are in the unit of the phenotype analysed and should be interpreted as a slope coefficient where the value of the phenotype changes for each unit copy of the allele present, so the *β* value should be doubled when comparing diploid homozygous individuals. For instance, *β* = 2 for SNP_x_ indicates an increase of four units of phenotype for the individual with the alternative allele of SNP_*x*_ in the homozygous state.

The *k*-mer GWAS was conducted following Voichek and colleagues^51^. The top 5% significant *k*-mers were mapped with bowtie 2 (v2.4.4)^91^ to the four Santo Antão assembled genomes (see section below). When plotting, we excluded *k*-mers with mapping quality of less than 24 against the TAIR10 genome.

### Chromosome 5 gbM peak analysis

To verify whether the peak at chromosome 5 in the gbM GWAS could be an artefact due to a signal from the deletion at *VIM2/VIM4* in CVI, we mapped the *k*-mers associated with gbM to four new CVI assemblies (PRJNA1112558) and Col-0 TAIR10 using Bowtie (parameter --all to return all mapping positions, 2 mismatches allowed). To assess the location of the *k*-mers relative to *VIM2, VIM4* and *VIM3* in each assembly, we blasted them using blastn (v2.2.27)^92^. We calculated the linkage disequilibrium between the top SNP (Chr5:15,047,549) and the surrounding SNPs using plink91 (v1.90b6.26)^93^. Linkage disequilibrium between SNP chr5:15047549 and the *VIM2/4* deletion was calculated among the CVI accessions in R.

### Phenotypic association analyses

To evaluate the phenotypic impact of the three derived methylation variants (*CMT2*_*stop*_, *FBX5*_*stop*_ and *VIM2/4*_*del*_), we analysed their association with previously published trait data for flowering time and WUE (measured via δ^13^C) from the Santo Antão accessions^48,63^. Statistical associations were tested using standard linear models.

As the *FRI*_*K232X*_ loss-of-function mutation was previously established as the primary driver of early flowering and adaptive drought escape in the Santo Antão population^48^, we incorporated the *FRI* genotype (ancestral versus derived) as a fixed-effect covariate when assessing flowering time. This allowed us to determine the independent phenotypic effects of the derived methylation variants while controlling for the background *FRI* mutation. The linear model for flowering time was constructed as follows:

$$y={\beta }_{0}+{\beta }_{1}{X}_{\rm{variant}}+{\beta }_{2}{X}_{\rm{FRI}}+\epsilon$$

where y is the flowering time (days to flowering), ${{{X}}}_{{\rm{variant}}}$ is the genotype of the methylation variant (ancestral or derived), ${{{X}}}_{{\rm{FRI}}}$ is the genotype at the *FRI*_*K232X*_ locus, ${{{\beta }}}_{{{1}}}$ represents the fixed-effect estimate (effect size) of the derived methylation variant, ${{{\beta }}}_{{{2}}}$ represents the effect of the *FRI* covariate and ${{\epsilon }}$ is the residual error.

For WUE (δ13C), we utilized a univariate linear model without the covariate

$$y={\beta }_{0}+{\beta }_{1}{X}_{\rm{variant}}+\,\epsilon$$

where ${{y}}$ is the WUE (δ^13^C), ${{{X}}}_{{\rm{variant}}}$ is the genotype of the methylation variant and ${{{\beta }}}_{{{1}}}$ is the fixed-effect estimate of the derived allele. For both models, we extracted the effect size ${{\beta }}$, s.e.m. and significance (*P*value derived from the *t*statistic) for the derived alleles to quantify their association with alternative drought adaptation strategies.

### Marginal genealogical tree with RELATE

We used RELATE v1.1.4^65^ to infer the genealogical trees for the derived allele variants of *CMT2*, *FBX5* and *VIM2*. We used bcftools^94^ v1.9 to filter the VCF file for quality, remove non-biallelic SNPs, retain segregating sites and filter out missing data with the following execution <bcftools view -m2 -M2 -v snps –min-ac=1 -i ‘MIN(FMT/DP) > 3 & MIN(FMT/GQ) > 25 & F_MISSING = 0’>. Within RELATE, we used the command RelateFileFormats (using --mode ConvertFromVcf) to convert the VCF file into haplotype and sample files. For the deletion variation in *VIM2*, we introduced a SNP variant (Chr1:24,586,731) as a proxy for the deletion region into the ‘hap’ file. We ran RELATE under a haploid model for chromosomes that contain the mentioned variants (using –mode All) and defined parameters of recombination map (-map), mutation rate (-m), and coalescence rates (--coal). For the ‘-m’ parameter, the estimated mutation rate (7 × 10^−9^) for *A. thaliana*^95^ was corrected for the per cent missing data in 1-Mb sliding windows every 50 kb across the entire genome. We calculated the mean of the corrected mutation rate for the 1-Mb sliding windows carrying the mutation and obtained 2.142 × 10^−9^ for both *CMT2* (Chr4:10,707,974) and *FBX5* (Chr2:18,513,626) variants, and 2.2131 × 10^−9^ for *VIM2* (Chr1:24,586,731) variant. We used a published recombination map^96^ and corrected for the estimated outcrossing rate of 5% estimated in natural populations^97^ by dividing the genetic distances by 20. We used the genome-wide coalescence rates (--coal) inferred previously^48^ for the Santo Antão population to infer the local genealogies for the studied variants. We set the generation time ‘--years_per_gen’ to 1 year. To produce genealogical trees with CIs for the estimated ages based on 200 samples from the MCMC (derived using SampleBranchLengths.sh --format a, and using default settings), we used the script TreeViewSample.sh, with 10× *N* steps (*N* is the number of haplotypes) and 1,000 burn-in iterations.

### Inference of selection coefficients

We used CLUES^66,98^ to infer selection coefficient and the frequency trajectory for the derived allele variants of CMT2 (Chr4:10,707,974), FBX5 (Chr2:18,513,626) and VIM2 (Chr1:24,586,731). CLUES uses importance sampling over trees generated in RELATE to produce a posterior distribution of trees from which a frequency trajectory can be inferred. From the output from RELATE, we used the command ‘./SampleBranchLengths.sh --format b’ to obtain 200 samples from the MCMC. We obtained estimates of the posterior distributions of allele frequencies over time using a recessive model (--dom 0): < inference.py --tCutoff 5000 --coal relate.popsize.coal --sMax 1 -df 100 --dom 0 >. For the ‘--popFreq’ parameter, we set the allele frequency as follows: CMT2 and FBX5, 0.33 and VIM2, 0.468. We inferred selection coefficient for CMT2, FBX5 and VIM2 variants in one-time epoch (0–1.5 kya) between the present day and the time in the past when the variant arose.

### Selective sweep and population genetic differentiation analyses

To quantify genome-wide differentiation between populations, we calculated the pairwise fixation index (*F*_ST_). We employed the weighted estimator of Weir and Cockerham (1984)^99^ as implemented in VCFtools^100^. The genome scan was conducted using overlapping sliding windows of 10 kb with a step size of 1 kb (--fst-window-size 10000 --fst-window-step 1000) to capture regional patterns of divergence. Windows falling within the top 5% of the empirical *F*_ST_ distribution were identified as significant outliers, representing putative genomic footprints of differentiation between populations.

We identified genome-wide signatures of positive selection using the composite likelihood method implemented in RAiSD v2.8^101^. This approach calculates the *μ*statistic, which integrates changes in genetic diversity, the site frequency spectrum and linkage disequilibrium to detect sweeps. The analysis was performed on haploid data (-y 1) using a sliding window of 50 SNPs (-w 50). To ensure robustness, we disabled missing data imputation (-M 0), relying strictly on observed genotypes. Regions with *μ* statistic values in the top 5% of the genome-wide distribution were considered candidate regions for positive selection.

### Whole-genome assemblies

Four accessions from Santo Antão (Cvi-0, S1-1, S5-10 and S15-3) were sequenced and assembled using long-read sequencing by the Max Planck Genome Centre in Cologne (Germany). In brief, the genomic DNA was extracted from pooled leaf tissue from the same genotype. DNA fragments above 30 kb were selected with BluePippin (Sage Science). The libraries were prepared using Ligation Sequencing Kit 1D (Oxford Nanopore Technologies, SQK-LSK109) and sequenced using the GridION X5 platform (Oxford Nanopore Technologies). De novo assemblies were generated with miniasm version 0.3^102^ and minimap version 2-2.17^103^. The resulting draft assemblies were first polished twice with racon (v1.4.10)^104^ using the raw Nanopore reads and then ten more times with pilon (v1.23)^105^ using the corresponding Illumina short reads. We oriented and scaffolded the contigs using the reference genome assembly (TAIR10). To prevent mapping of Illumina short reads to anchoring points of the contigs, we inserted 1,000 Ns between the contigs. Nanopore reads mapping the *VIM2/4* region are present in NCBI under accession code PRJNA1112558.

### Whole-genome bisulfite sequencing

Rosette leaves were harvested 20–25 days after germination at stage 4–8 true leaves. Collection was performed between Zeitgeber (ZT) 3 and 6 for whole-genome bisulfite sequencing. Samples were directly flash-frozen in liquid nitrogen after collection. Whole-genome bisulfite sequencing libraries were prepared as in ref. ^106^. Libraries were sequenced on HiSeq3000 in 150-bp single-end mode to generate about 7 million reads of data per library. Adaptors were trimmed using Cutadapt (v3.5)^107^ and mapped on TAIR10 reference genome with Bismark (v0.19.0) (parameters –N 1)^108^. The conversion efficiency was estimated using the levels of unconverted cytosines found in the chloroplast unmethylated genome. Levels of converted cytosines were higher than 99% in all cases. Cytosine reports from Bismark were imported in R and subsequent methylation analyses were performed with the methylKit package (v1.14.2)^109^. DMRs were defined with window size of 300 bp and an overlapping of window of 100 bp. Overlapping DMRs with a methylation difference >25% and a *q*value <0.01 were merged and their differential methylation levels were averaged. Although 7 million reads per library would be low for calling DMRs across individual genomes, our DMR analysis was conducted by aggregating across accessions carrying each state of the VIM2 allele to identify loci that were differentially methylated between the two VIM alleles.

To compare our data with the 1001GP analysis, we bisulfite sequenced the two outlier accessions Dör-10 (seqID 5856, Sweden) and UKID116 (seqID 5822, the UK), which showed high and low gbM, respectively^45^ and Col-0, which exhibits an intermediate value. Levels of gbM for UKID116 and Dör-10 in our data diverge from only 1–2% of the values from the 1001GP data using our analysis pipeline. The gbM levels of these two accessions encompasses the values of gbM found of the Moroccan accessions, with Cvi-0 being close to UKID116 and Col-0 showing intermediate values, as previously reported. Summary statistics of the whole-genome bisulfite sequencing data are presented in Supplementary Table 1.

### Whole-genome transcriptome sequencing

We generated leaf transcriptomes for 97 Santo Antão accessions, including 11 accessions with three biological replicates. Rosette leaves from 4-true leaves (20 days after germination) were collected between ZT3 and ZT6 and flash-frozen into liquid nitrogen. Samples were ground in 2 ml Eppendorf tubes containing one tungsten carbide ball in a TissueLyser II (Qiagen). An aliquot of about 20 mg of powder was transferred into a 96-well plate, and total RNA was prepared with the NucleoMag 96 RNA kit (Macherey Nagel). Libraries were prepared with the NEBNext Ultra Directional RNA Library Prep Kit for Illumina sequencing (New England Biolabs). Approximately 7 million reads of 150-bp single-end reads were generated on the Illumina sequencer HiSeq3000. The adaptors were trimmed using Cutadapt (v3.5) (parameters -m 20 -q 35)^107^. Reads were mapped on TAIR10 reference genome and Araport11 gene annotation using HISAT2 (v2.2.0)^110^. The read count was performed with HTSeq (v0.12.4)^111^. Differential expression analysis was performed in R using the DESeq2 package (v1.28.1)^112^. Biological replicates of the 11 accessions cluster tightly on the principal component analysis for gene expression, indicating that environmental effects on gbM within the growth chamber were minimum (Supplementary Fig. 4). Details of the RNA-sequencing libraries are presented in Supplementary Table 7.

### *VIM2/4* deletion genotyping

To detect the *VIM2/VIM4* deletion in the Cape Verde accessions sequenced, we could not rely on the broken paired-end reads due to the high homology between *VIM2* and *VIM4*. Instead, we defined a minimum sequencing coverage of 50 reads at the defined location of the deletion (chr1:24,586,731–24,589,471) below which we consider an accession as having the *VIM2/VIM4* deletion. The read count was performed with Samtools (v1.9).

### TE expression analysis

Expression levels of TEs was measured using RepEnrich2^113^ using the TAIR10 TE annotation available on https://www.arabidopsis.org/. The RepEnrich2_subset.py script was run using the mapq parameter 30. The raw read count for each TEs was processed in R using the DESeq2 package (v1.28.1) and differential expression was calculated by grouping accessions by *CMT2* or *FBX5* allele using a significance threshold of 5%. Permutation for the number of TEs with log_2_FC superior to 2 was performed by permutating alleles randomly within populations and evaluating how many TEs are differentially expressed when allele status is attributed randomly. The permuted distribution was compared to the observed value and the *P* value was derived using the formula (*B* + 1)/(*M* + 1), where *B* is the number of permutations in which a value greater or equal than the observed value and *M* is the total number of permutations sampled.

### TE transposition analysis

An estimate of TE transposition in each accession was calculated using the percentage of WGS reads mapping to the TAIR10 TE annotation (31,189 TEs) using Bowtie (default parameters). Accessions with higher percentages of reads mapping at TEs are assumed to have a higher TE load and therefore likely to have a higher transposition activity. Accessions were grouped by *CMT2* and *FBX5* alleles and the difference in TE mapping rate was tested using a Wilcoxon test.

### Production and analysis of CRISPR lines

CRISPR–Cas9 transgenic lines for *FBX5* were generated using an egg-cell promoter-based system^114^ with details available in ref. ^115^ (primers listed in Supplementary Table 13). Transformed T1 plants in the S7-B5 background (CVI accession with *FBX5*_*ref*_ allele) were recovered from hygromycin plates and transplanted in soil. Mutations at the target sites were verified using Sanger sequencing. We also generated high-coverage (ranging from 59.7× to 81.1×) short-read genomic data for the CRISPR lines to investigate the potential for off-target mutations. We extracted DNA with the DNeasy Plant Mini Kit (Qiagen, 69106) and prepared the libraries using NEBNext Ultra II FS DNA Library Prep (Illumina, New England Biolabs). Sequencing was conducted on an Illumina HiSeq3000 platform (Illumina, NEB) with 68–82 million reads (2× 150 bp) (Novogene). We examined potential off-target mutations as in ref. ^116^. In brief, the resulting reads were mapped to TAIR10 with bwa (version 0.7.15) using the mem algorithm. We used GATK to call SNPs and short indels and predicted potential off-target sites with Cas-OFFinder (v3.0.0)^117^. We used a maximum of five mismatches, SpCas9 from *Streptococcus pyogenes* (5′-NGG-3′) as CRISPR–Cas-derived RNA-guided endonuclease and TAIR10 as a target reference genome. Finally, we examined evidence for overlap between predicted off-target sites and SNPs and indels. We found no evidence of off-target mutations in the CRISPR lines. T3 seeds of three independent *FBX5* CRISPR–Cas9 transgenic lines were used for whole-genome bisulfite sequencing.

### Reporting summary

Further information on research design is available in the Nature Portfolio Reporting Summary linked to this article.

## Supplementary information


Supplementary InformationSupplementary Figs. 1–7.
Reporting Summary
Supplementary Tables


## Data Availability

Raw sequencing read data for whole-genome bisulfite sequencing and RNA-sequencing were deposited in NCBI under the accession codes PRJNA612437 (https://dataview.ncbi.nlm.nih.gov/object/PRJNA612437?reviewer=bd1t2mijqq0n460nr58eso2co1) and PRJNA874864, respectively. The Nanopore whole-genome assemblies of the SA accessions S1-1, S15-3, S5-10 and Cvi-0 (region Chr1:24,400,000–24,800,000) are available in PRJNA1112558 (https://dataview.ncbi.nlm.nih.gov/object/PRJNA1112558?reviewer=8tili8lhu4irrubf98lvc1nbas). WGS of CRISPR lines for off-target analysis are available in PRJNA1112441 (https://dataview.ncbi.nlm.nih.gov/object/PRJNA1112441?reviewer=biuem2b3kbp7jt2eqbf5emis4i).

## Code availability

Scripts of the bioinformatics analyses are available via GitHub at https://github.com/johanzi/scripts_cvi_methylation_variants and via Zenodo at https://doi.org/10.5281/zenodo.20757903 (ref. ^118^).
