# Supplementary material for: A VIM2/4 deletion, and premature truncations of CMT2 and FBX5, drive DNA methylation changes after colonization of a novel habitat
Source: Nat Plants. 2026 Aug 3;12(8):1469–84. doi: 10.1038/s41477-026-02352-2 (PMC13481252; doi:10.1038/s41477-026-02352-2)
Supplement: Supplementary file 1 [file 41477_2026_2352_MOESM1_ESM.pdf]

nature plants

Supplementary information https://doi.org/10.1038/s41477-026-02352-2

A VIM2/4 deletion, and premature truncations of *CMT2* and *FBX5*, drive DNA methylation changes after colonization of a novel habitat

In the format provided by the authors and unedited

Supplementary Information for:

A VIM2/4 deletion, and premature truncations of *CMT2* and *FBX5*, drive DNA methylation changes after colonization of a novel habitat

Johan Zicola, Emmanuel Tergemina, Ahmed F. Elfarargi, Mehmet Göktay, Célia Neto, Robert J. Schmitz, and Angela M. Hancock

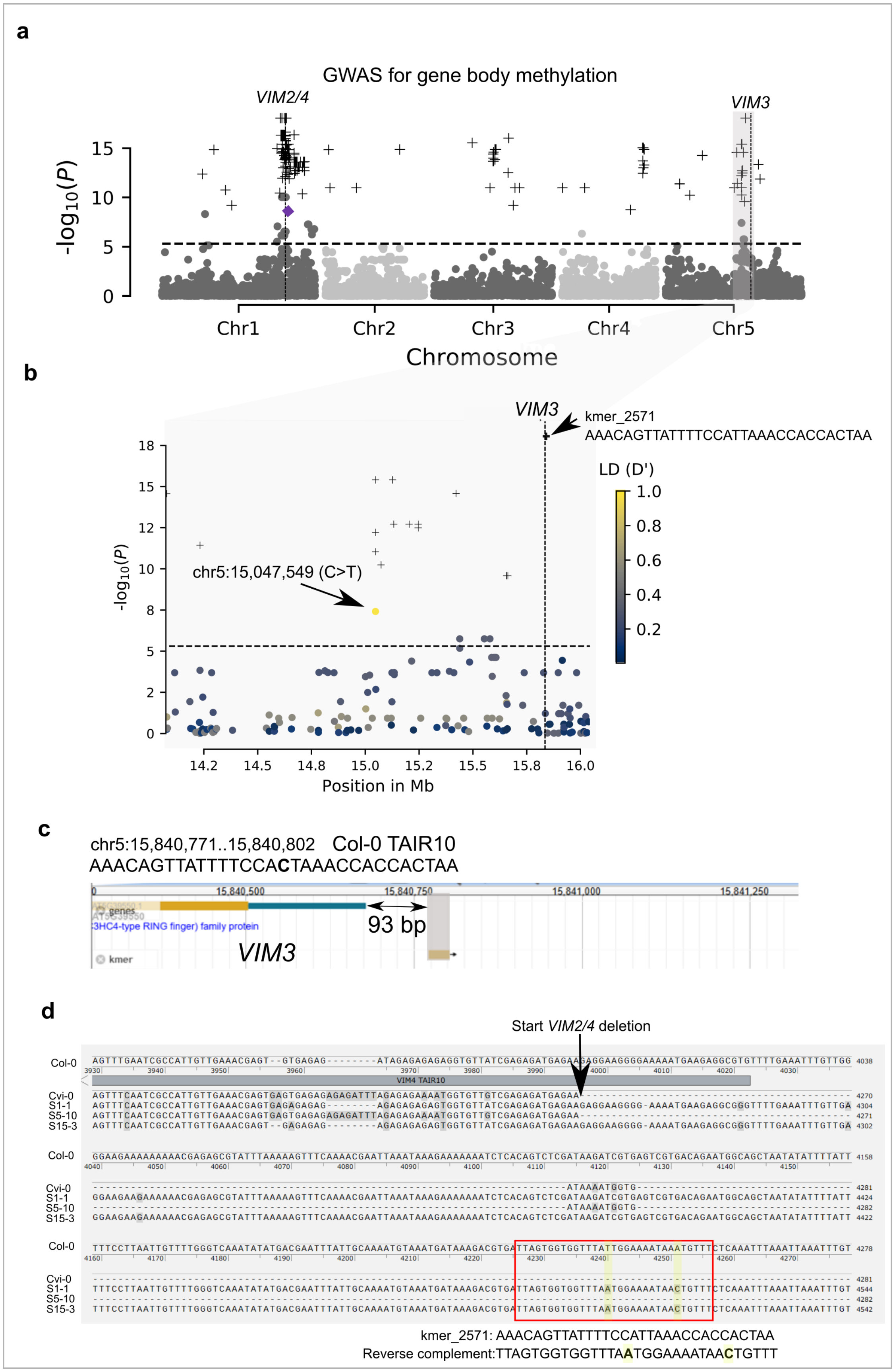
a
GWAS for gene body methylation
VIM2/4
VIM3
-log10(P)
Chromosome
Chr1
Chr2
Chr3
Chr4
Chr5
b
VIM3
kmer_2571
AAACAGTTATTTTCCATTAAACCACCACTAA
LD (D')
chr5:15,047,549 (C>T)
Position in Mb
c
chr5:15,840,771..15,840,802 Col-0 TAIR10
AAACAGTTATTTTCCACTAAACCACCACTAA
93 bp
VIM3
d
Start VIM2/4 deletion
Col-0
Cvi-0
S1-1
S5-10
S15-3
kmer_2571: AAACAGTTATTTTCCATTAAACCACCACTAA
Reverse complement:TTAGTGGTGGTTTAATGGAAAATAACTGTTT

Supplementary Fig. 1 | kmer-GWAS signal for the VIM3 region with gbM aligns to both the VIM2/3 and VIM3 genomic regions in Col-0. *a,* Manhattan plot for the gbM GWAS in Santo Antão using *k*-mers. *k*-mers are denoted by crosses. Bonferroni threshold is indicated by the dashed line. The most significant SNP is annotated with a purple diamond (~ 29.8 kb downstream, of *VIM2*). Positions of *VIM2/4* and *VIM3* are indicated by vertical dashed lines. b, Magnification of the Manhattan plot at the region 14-16Mb of chromosome 5 which contains *VIM3*. The location of the most significant kmer (kmer_2571) and *VIM3* is indicated by a red line. Colors indicate linkage disequilibrium (D') to the top SNP (chr5:15,047,549) indicated with an arrow. c, Position of the kmer_2571 in Col-0 assembly (mismatch indicated in bold) within *VIM3* promoter (gray area). d, Multiple alignment of the VIM4 promoter region for Col-0 (TAIR10) and four CVI assemblies: Cvi-0, S1-1, S5-10, and S15-3. The start of the deletion (present in Cvi-0 and S5-10) is indicated by an arrow. The mapping position of kmer_2571 on the VIM4 promoter is indicated by a red square and the two mismatches found in Col-0 are indicated by yellow boxes. The reverse complement sequence of the kmer is shown with mismatch locations in Col-0 highlighted in yellow. Nucleotides in gray indicate polymorphisms compared to Col-0 and hyphens indicate deleted nucleotides. The picture was taken from SnapGene software (from Dotmatics; available at snapgene.com).

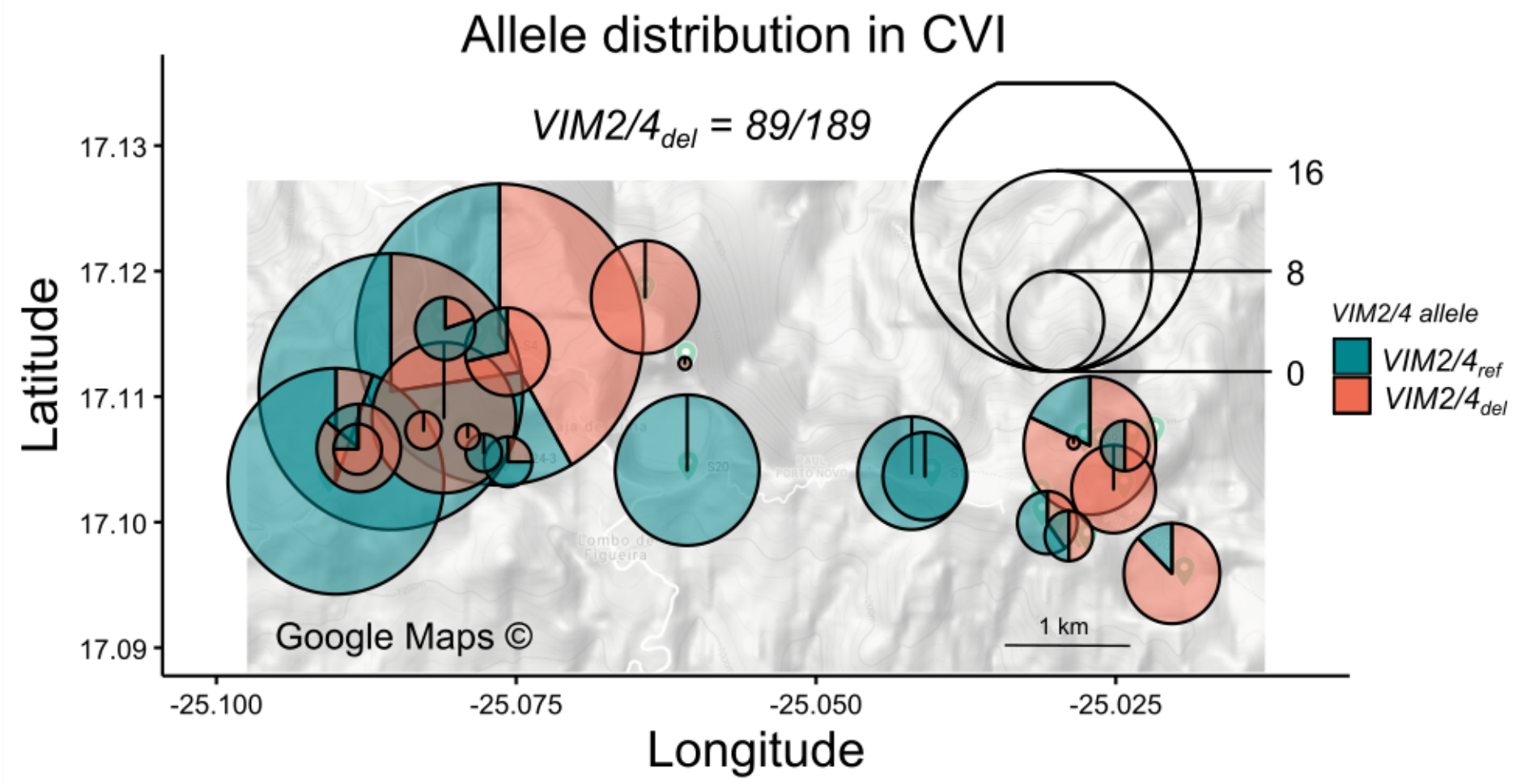

Supplementary Fig. 2 | *VIM2/4* deletion distribution within the 24 populations of 189 Arabidopsis accessions collected in Santo Antão. Spatial distribution of *VIM2/4*$_{del}$ in Santo Antão. Pie charts show the frequencies of *VIM2/4* alleles across sampling sites, with size representing the number of individuals per sampling site. Source map: Google Maps.

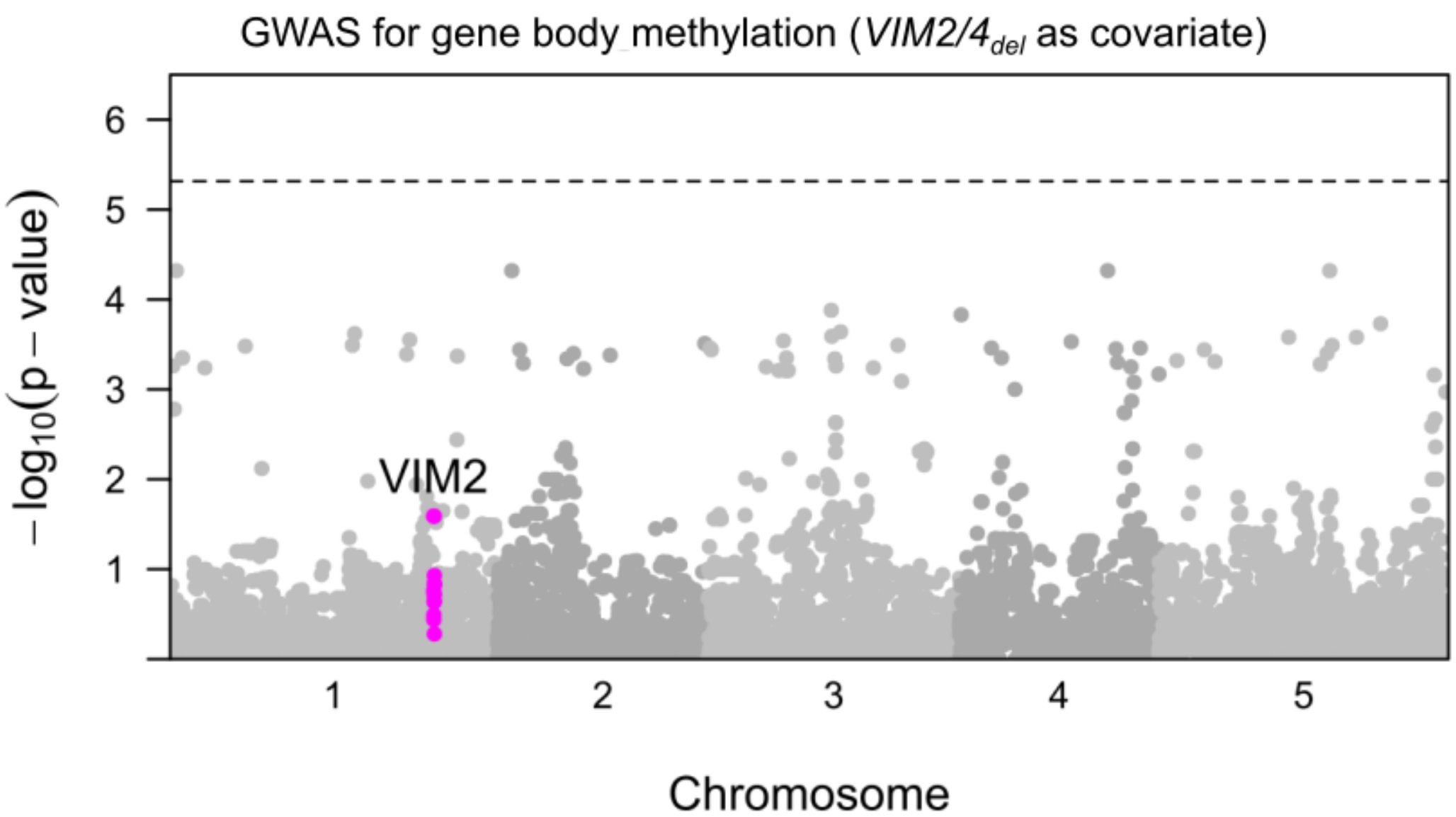

Supplementary Fig. 3 | Manhattan plot of GWAS for gbM with the *VIM2/4* deletion set as a covariate. SNPs surrounding *VIM2* (+/- 50 kb) are highlighted in purple to visualize the *VIM2* location on the Manhattan plot. 10,318 SNPs were analyzed with a minor allele frequency (MAF) threshold of 5%. Bonferroni threshold is indicated by a dashed line.

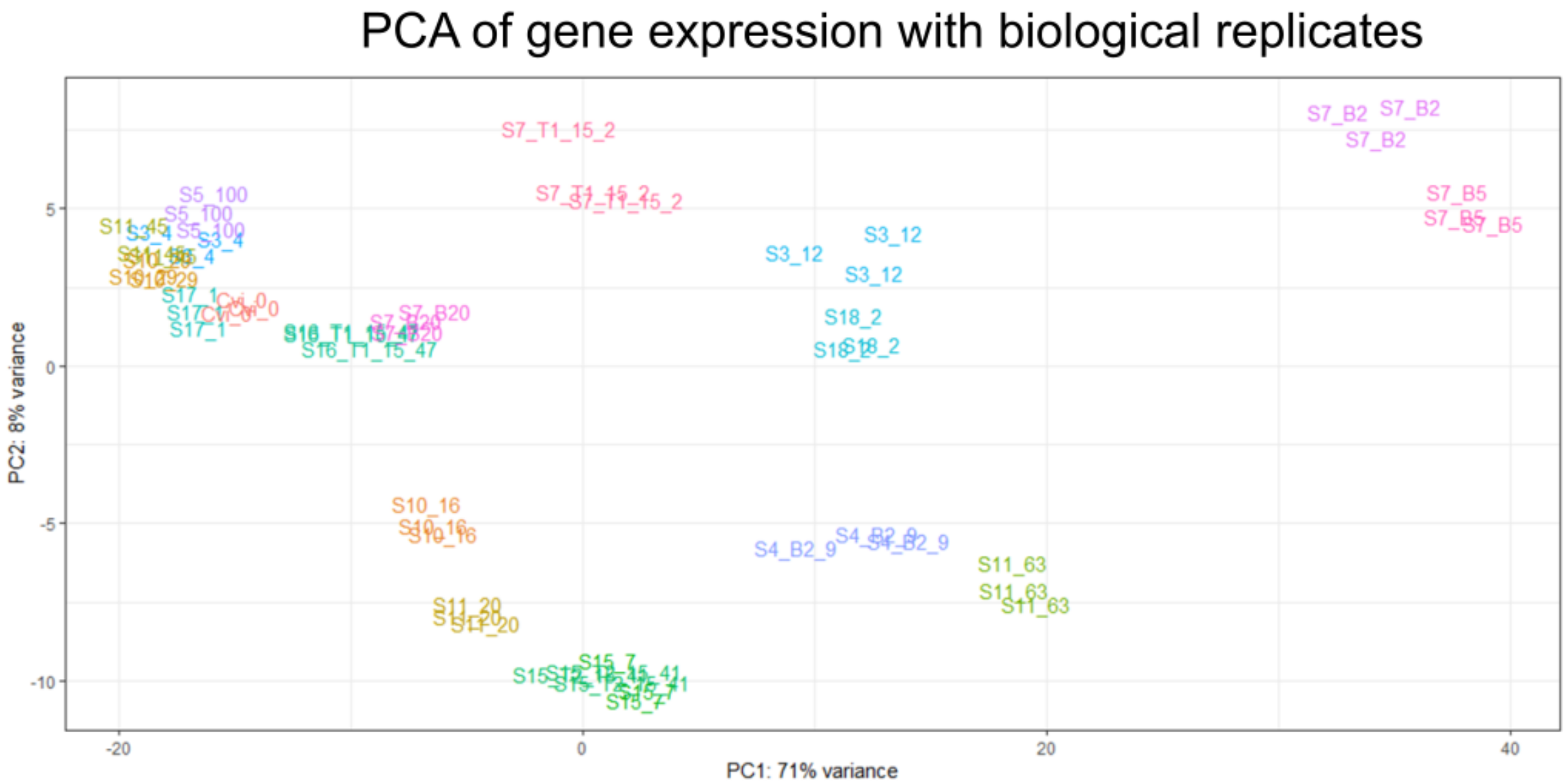

Supplementary Fig. 4 | PCA of gene expression for the twenty CVI accessions with biological replicates. 19 accessions have 3 biological replicates and 1 accession (S4-B3-14) has 2 biological replicates. 59 samples in total are represented and accessions are color-coded.

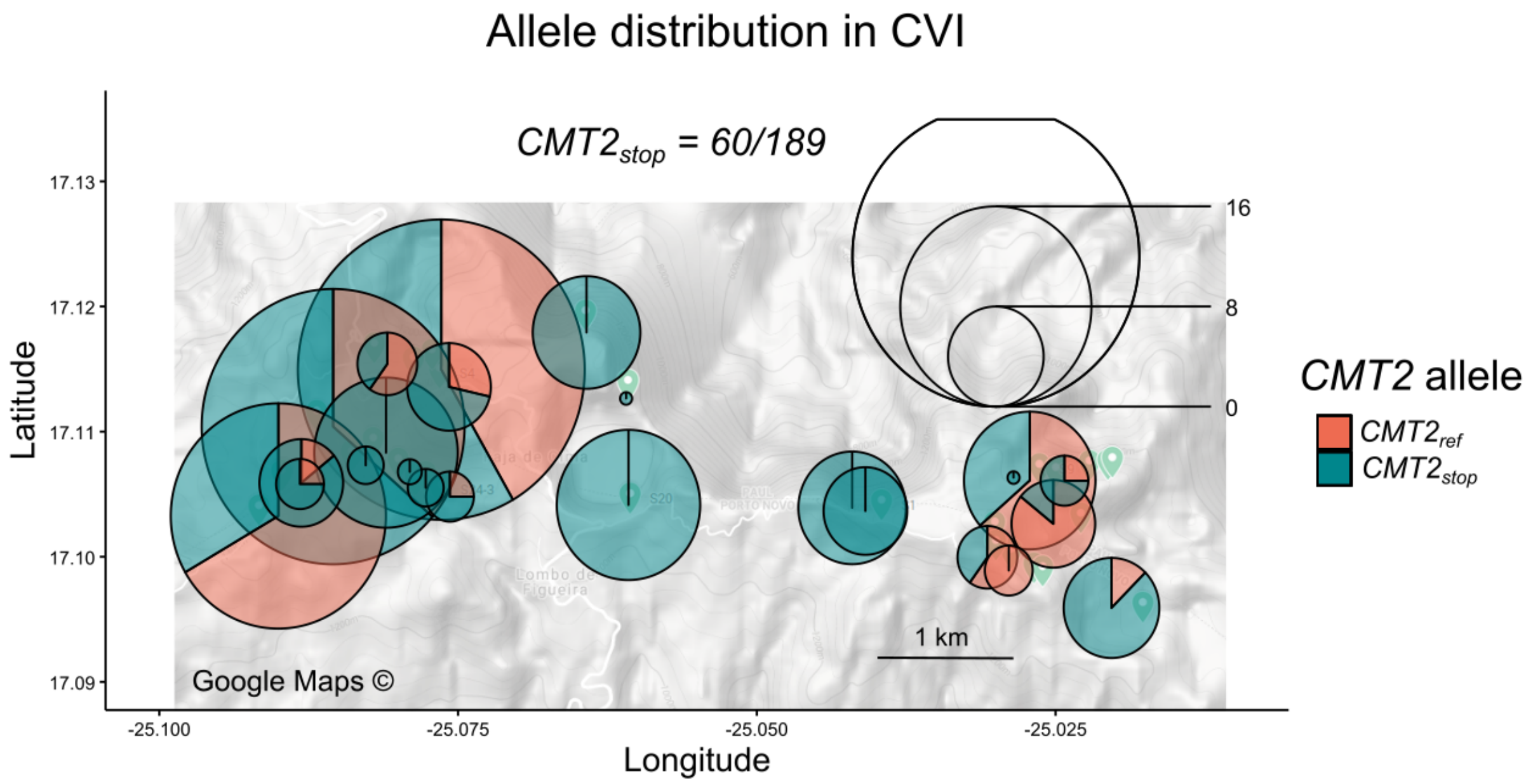

Supplementary Fig. 5 | *CMT2* allele distribution within the 24 populations of 189 Arabidopsis accessions collected in Santo Antão. Spatial distribution of *CMT2*$_{stop}$ in Santo Antão. Pie charts show the frequencies of *CMT2* alleles across sampling sites, with size representing the number of individuals per sampling site. Source map: Google Maps.

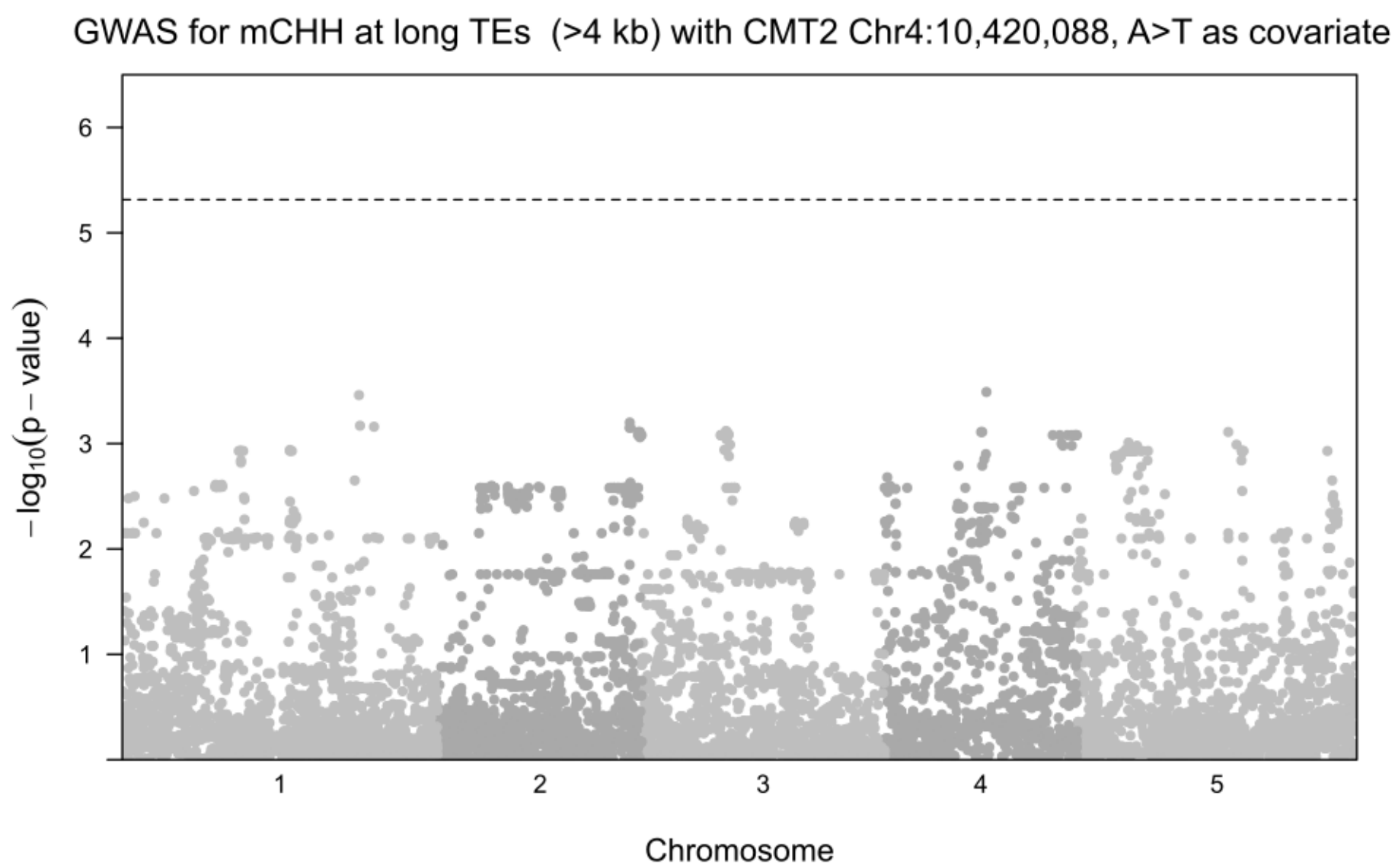

Supplementary Fig. 6 | GWAS for mCHH at long TEs (>4 kb) using the SNP causing a stop premature codon at CMT2 (Chr4:10,420,088, A>T) as covariate. 10,318 SNPs were analyzed with a minor allele frequency (MAF) threshold of 5%. Bonferroni threshold is indicated with the dotted line.

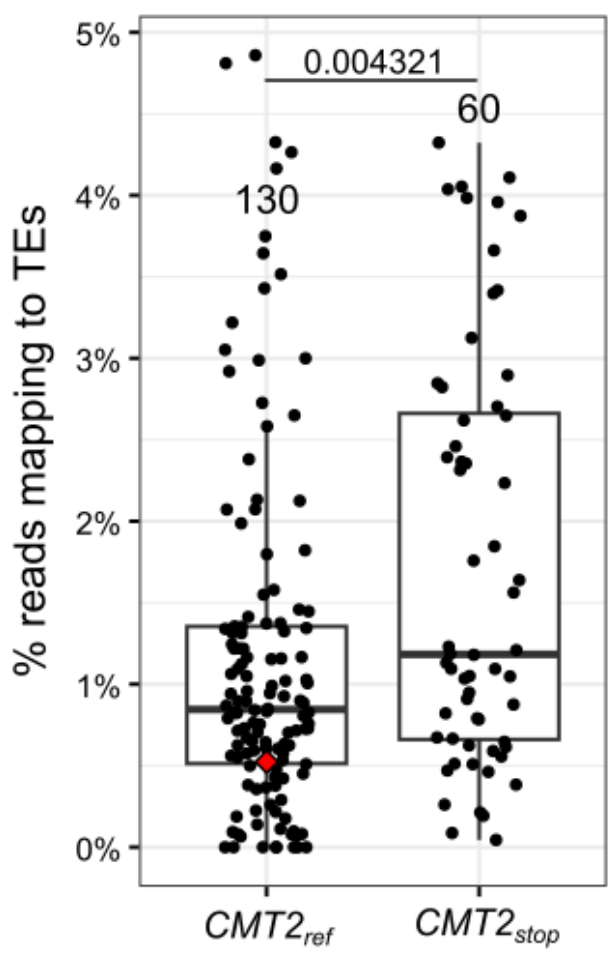

Supplementary Fig. 7 | Percentage of TE-mapped WGS reads in the 190 SA accessions. Paired-end read data were mapped onto the TE TAIR10 annotation (31,189 TEs) allowing two mismatches. Then, the percentage of TE-mapped reads relative to all reads was calculated (% reads mapping to TEs) for each allele. Cvi-0 is highlighted by a red diamond. Number of accessions in each group is indicated in the plot. The two groups were compared with the non-parametric Wilcoxon test and the p-value is indicated in the plot.

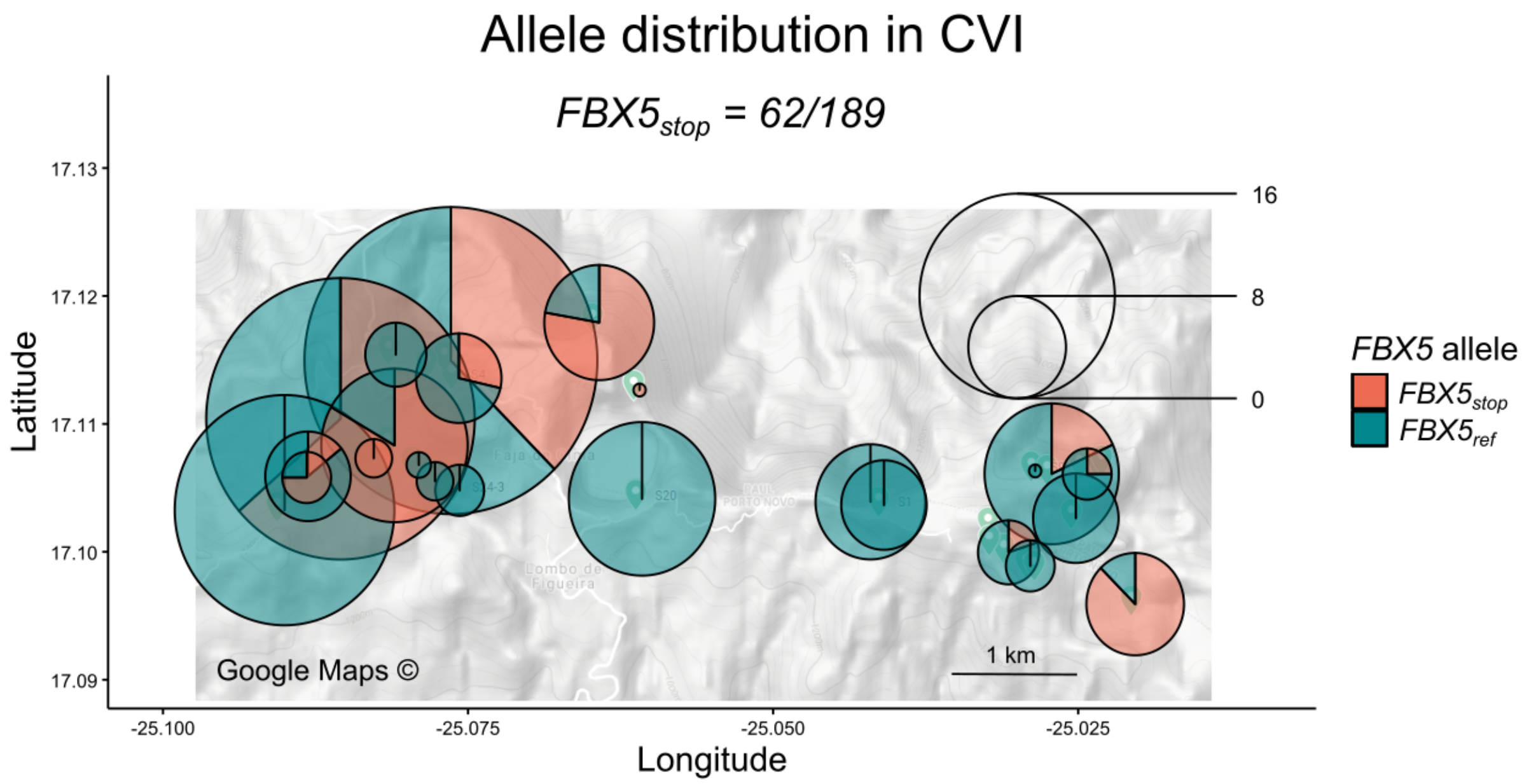

Supplementary Fig. 8 | *FBX5* allele distribution within the 24 populations of 189 Arabidopsis accessions collected in Santo Antão. Spatial distribution of *FBX5*$_{stop}$ in Santo Antão. Pie charts show the frequencies of *FBX5* alleles across sampling sites, with size representing the number of individuals per sampling site. Source map: Google Maps.

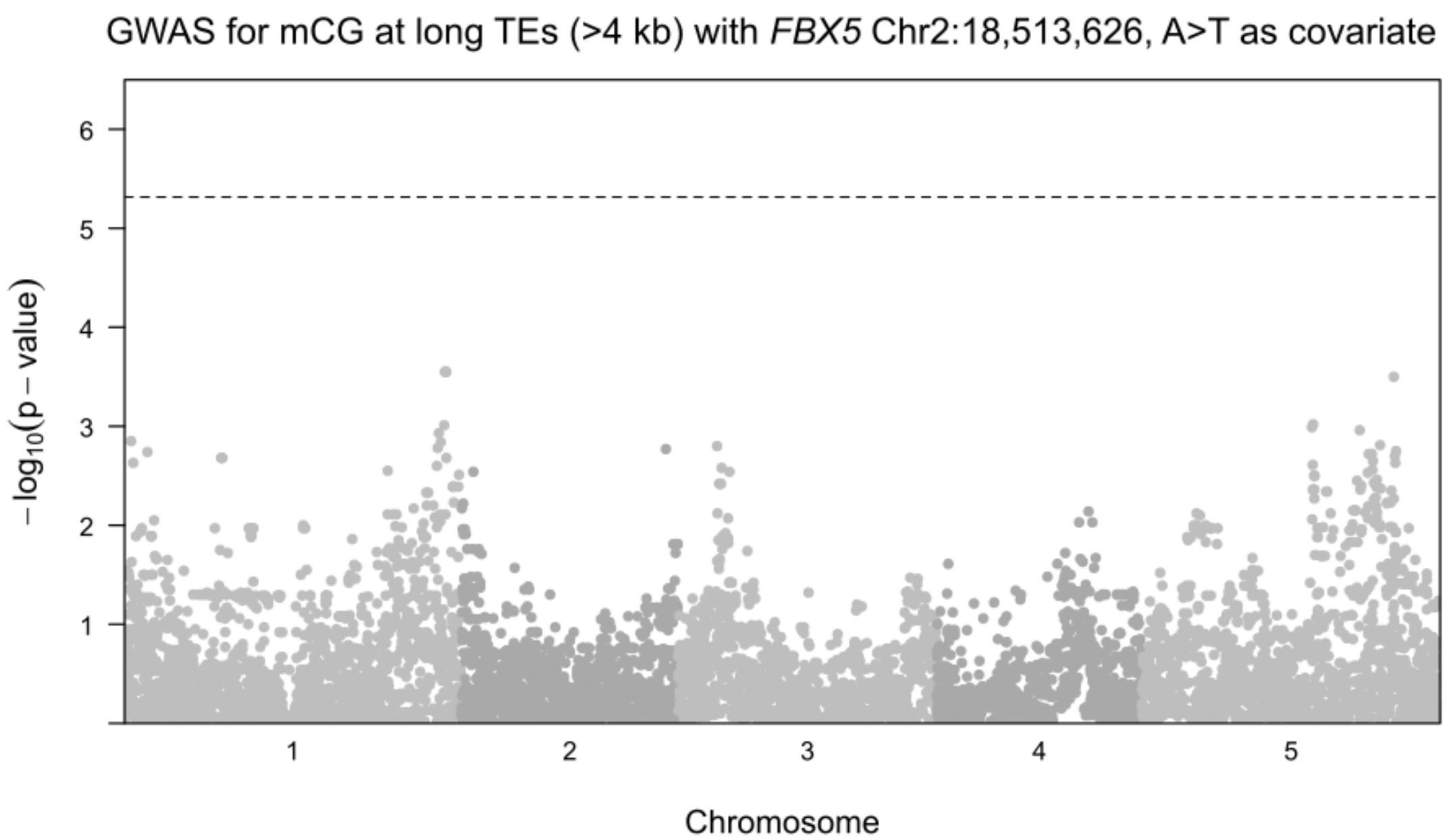

Supplementary Fig. 9 | GWAS for mCG at long TEs (4 kb) using the SNP causing a stop premature codon at FBX5 (Chr2:18,513,626, A>T) as covariate. 10,318 SNPs were analyzed with a minor allele frequency (MAF) threshold of 5%. Bonferroni threshold is indicated with the dotted line.

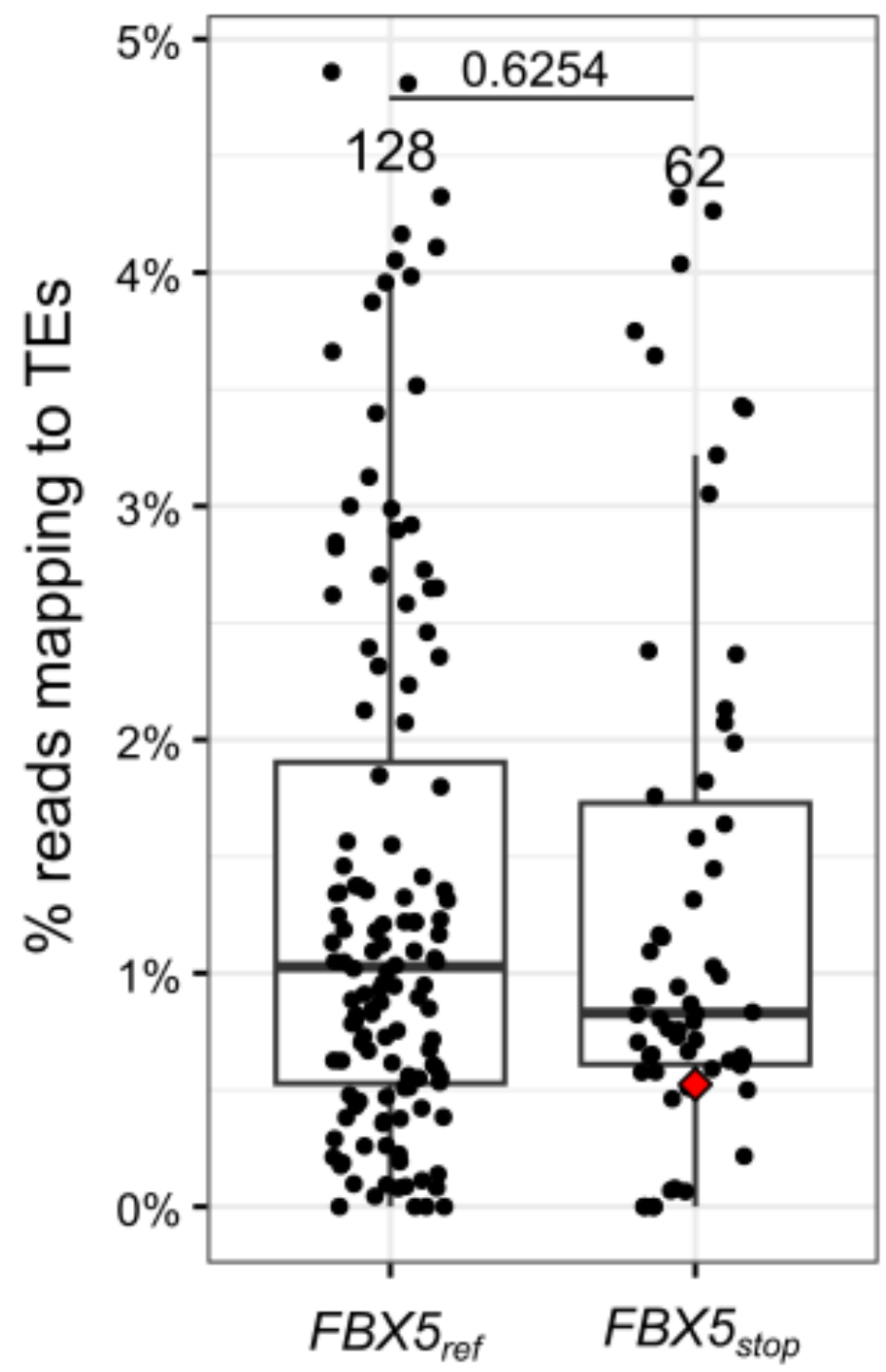

Supplementary Fig. 10 | Percentage of TE-mapped WGS reads in the 190 SA accessions. Paired-end read data were mapped onto the TE TAIR10 annotation (31,189 TEs) allowing two mismatches. Then, the percentage of TE-mapped reads relative to all reads was calculated (% reads mapping to TEs) for each allele. Cvi-0 is highlighted by a red diamond. Number of accessions in each group is indicated in the plot. The two groups were compared with the non-parametric Wilcoxon test and the p-value is indicated in the plot.

a

Genealogical tree and allele frequency trajectory of *VIM2/4*$_{del}$

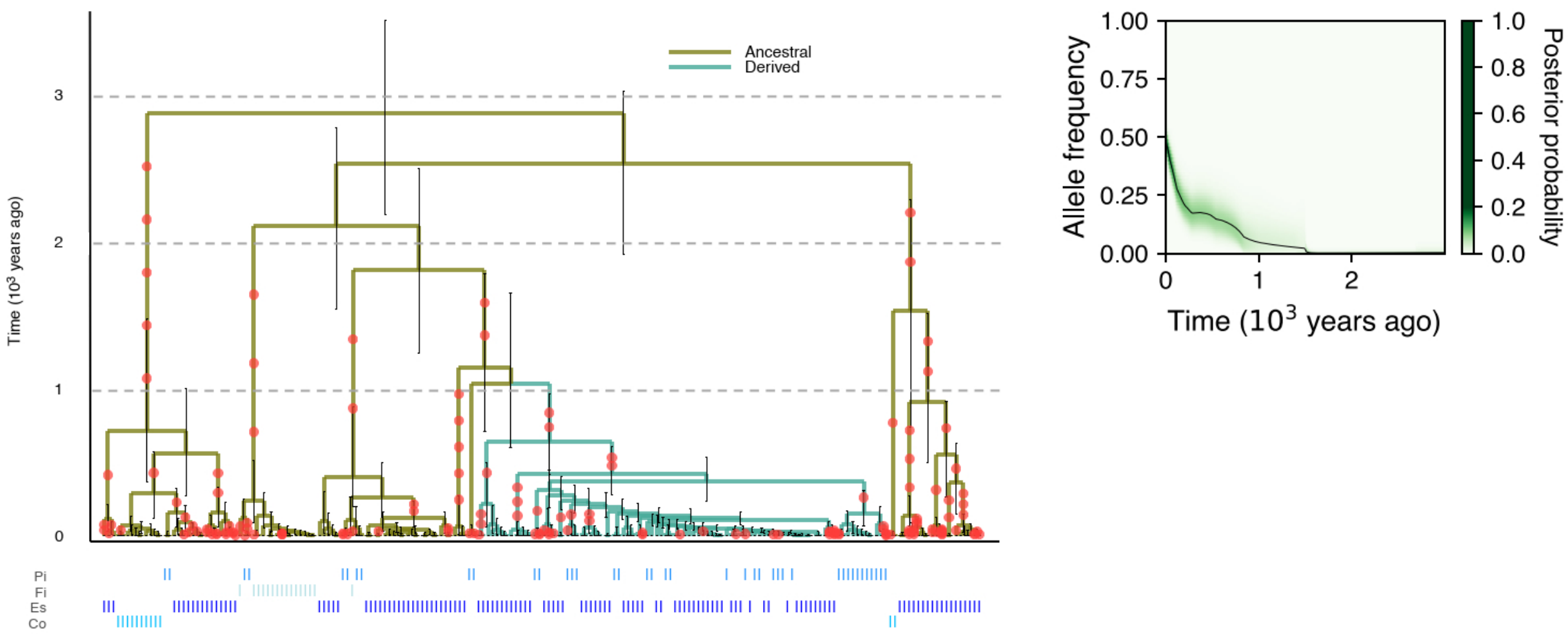

b

Genealogical tree and allele frequency trajectory of *CMT2*$_{stop}$

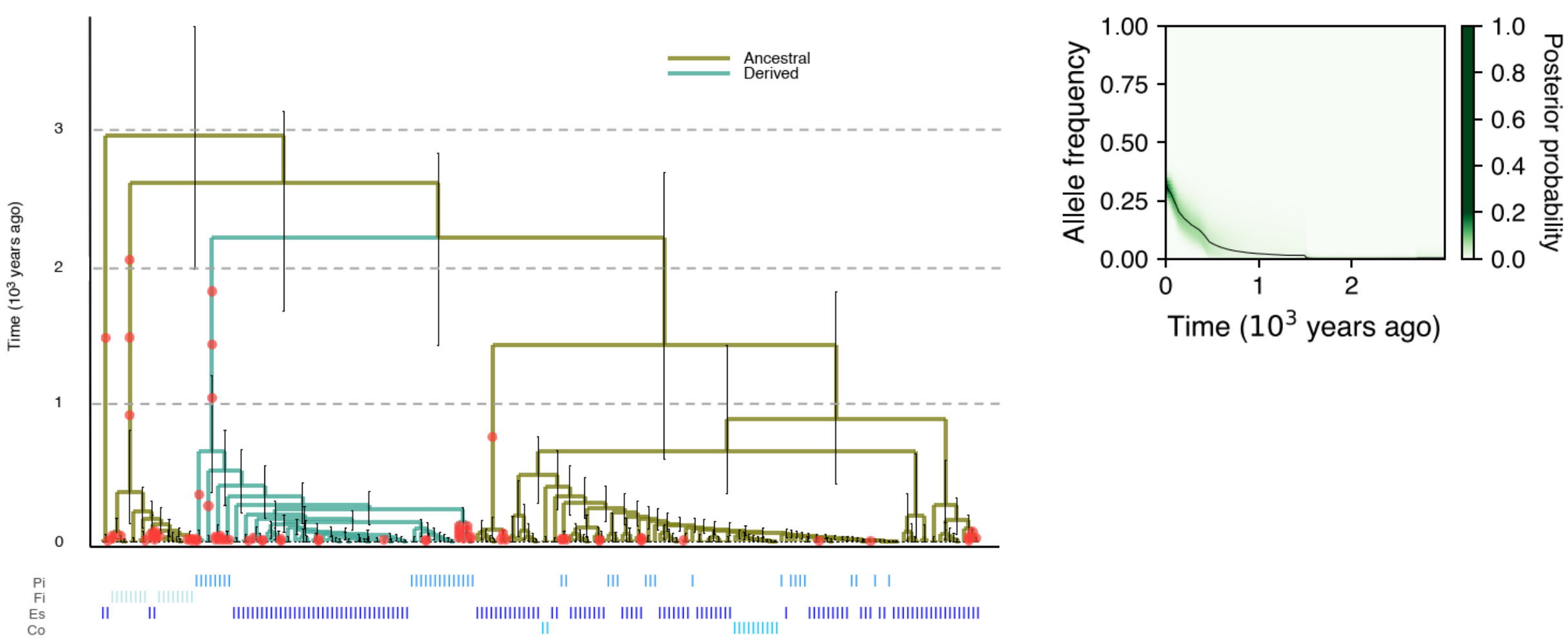

c

Genealogical tree and allele frequency trajectory of *FBX5*$_{stop}$

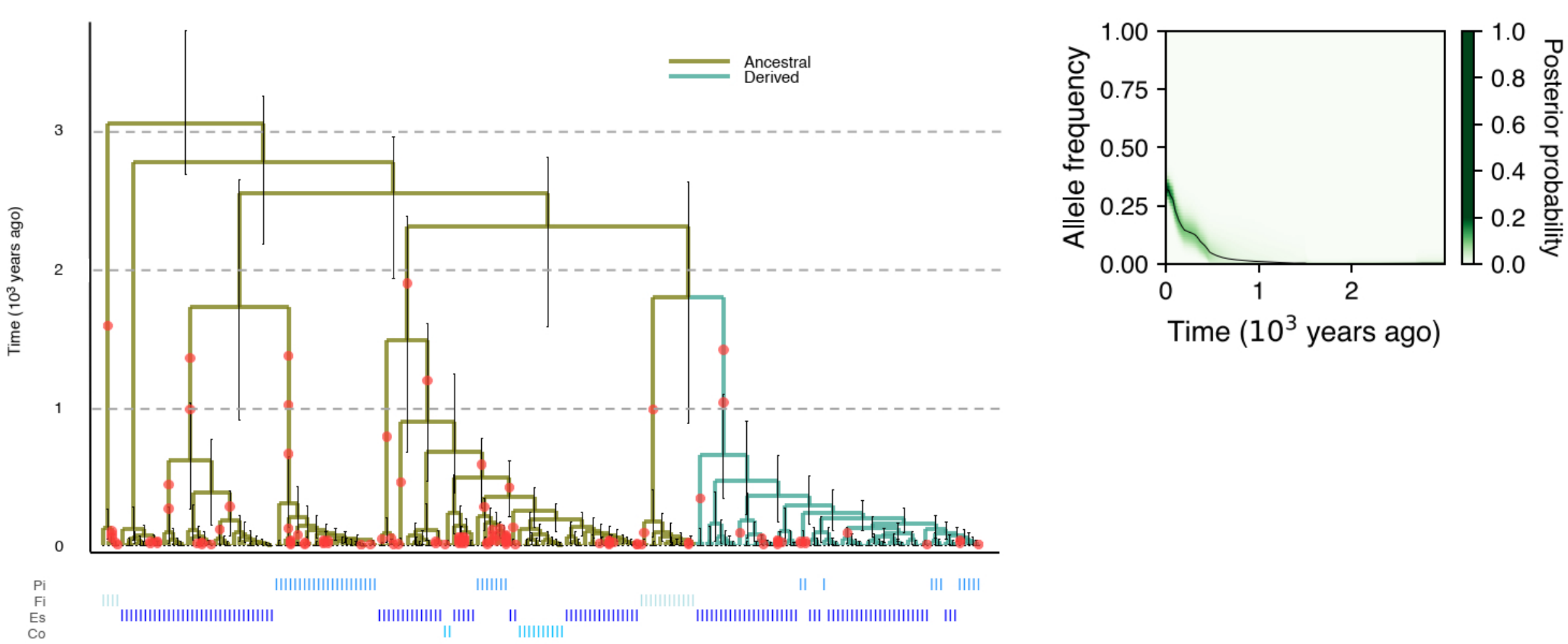

Supplementary Fig. 11 | Evolutionary history of the *VIM2/4$_{del}$* (a), *CMT2$_{stop}$* (b), and *FBX5$_{stop}$* (c) variants. For each panel, the left plot displays the inferred genealogical tree, and the right plot illustrates the corresponding allele frequency trajectory over time. In the tree plots (left), individual sampled haplotypes are arrayed along the x-axis, with ancestral and derived lineages colored in mustard and teal, respectively. The y-axis displays the estimated time to coalescence (in 10^3 years ago). Vertical black lines represent the 95% credible intervals (0.025 and 0.975 quantiles) of the posterior density for the coalescence times, while red dots denote discrete mutations mapped to their respective branches. The blue tick marks beneath each tree indicate the subpopulation origin of each sampled individual. In the allele trajectory plots (right), the solid dark line traces the posterior mean estimate of the derived allele frequency over time, bounded by a green shaded region that represents the full posterior probability distribution. Abbreviations for the Santo Antão subpopulations: Co, Cova de Paúl; Es, Espongeiro; Fi, Lombo de Figueira; Pi, Pico da Cruz.
