# Supplementary material for: A VIM2/4 deletion, and premature truncations of CMT2 and FBX5, drive DNA methylation changes after colonization of a novel habitat
Source: Nat Plants. 2026 Aug 3;12(8):1469–84. doi: 10.1038/s41477-026-02352-2 (PMC13481252; doi:10.1038/s41477-026-02352-2)
Supplement: Supplementary file 2 [file 41477_2026_2352_MOESM2_ESM.pdf]

nature portfolio

Corresponding author(s): Angela M. Hancock

Last updated by author(s): 12.06.2026

Reporting Summary

Nature Portfolio wishes to improve the reproducibility of the work that we publish. This form provides structure for consistency and transparency in reporting. For further information on Nature Portfolio policies, see our Editorial Policies and the Editorial Policy Checklist.

Statistics

For all statistical analyses, confirm that the following items are present in the figure legend, table legend, main text, or Methods section.

n/a	Confirmed	
☐	☒	The exact sample size (*n*) for each experimental group/condition, given as a discrete number and unit of measurement
☐	☒	A statement on whether measurements were taken from distinct samples or whether the same sample was measured repeatedly
☐	☒	The statistical test(s) used AND whether they are one- or two-sided<br>*Only common tests should be described solely by name; describe more complex techniques in the Methods section.*
☒	☐	A description of all covariates tested
☐	☒	A description of any assumptions or corrections, such as tests of normality and adjustment for multiple comparisons
☐	☒	A full description of the statistical parameters including central tendency (e.g. means) or other basic estimates (e.g. regression coefficient) AND variation (e.g. standard deviation) or associated estimates of uncertainty (e.g. confidence intervals)
☐	☒	For null hypothesis testing, the test statistic (e.g. *F*, *t*, *r*) with confidence intervals, effect sizes, degrees of freedom and *P* value noted<br>*Give P values as exact values whenever suitable.*
☒	☐	For Bayesian analysis, information on the choice of priors and Markov chain Monte Carlo settings
☒	☐	For hierarchical and complex designs, identification of the appropriate level for tests and full reporting of outcomes
☒	☐	Estimates of effect sizes (e.g. Cohen's *d*, Pearson's *r*), indicating how they were calculated

Our web collection on statistics for biologists contains articles on many of the points above.

Software and code

Policy information about availability of computer code

Data collection	No software was used for data collection.
Data analysis	Softwares used for the bioinformatics: Bismark (v0.19.0), Python3.5, GEMMA (v0.94), vcftools (v0.1.14), bcftools (v1.2), bwa (v0.7.15), bowtie (v1.2.2), bowtie 2 (v2.4.4), R (v3.3.0), DESeq2 package (v1.28.1), SRA tool kit (v3.41), GATK (v4.3.3), Cas-OFFinder (v3.0.0), FastQC (v0.11.9), HTSeq (v0.12.4), multiqc (v1.6), Cutadapt (v3.5), methylKit (v1.14.2), HISAT2 (v2.2.0), pilon (v1.23), racon (v1.4.10), miniasm-0.3, minimap2-2.17, RAiSD (v2.8), CLUES (v.2), RELATE (v1.1.4), blastn (v2.2.27), plink91 (v1.90b6.26). Scripts of the bioinformatics analyses are available on GitHub (https://github.com/johanzi/scripts_cvi_methylation_variants) and archived in Zenodo (https://doi.org/10.5281/zenodo.20757903).

For manuscripts utilizing custom algorithms or software that are central to the research but not yet described in published literature, software must be made available to editors and reviewers. We strongly encourage code deposition in a community repository (e.g. GitHub). See the Nature Portfolio guidelines for submitting code & software for further information.

Data

Policy information about availability of data

All manuscripts must include a data availability statement. This statement should provide the following information, where applicable:

- Accession codes, unique identifiers, or web links for publicly available datasets
- A description of any restrictions on data availability
- For clinical datasets or third party data, please ensure that the statement adheres to our policy

Raw sequencing read data for whole-genome bisulfite sequencing and RNA-seq were deposited in NCBI under the accession codes PRJNA612437 (https://dataview.ncbi.nlm.nih.gov/object/PRJNA612437?reviewer=bd1t2mijqq0n460nr58eso2co1) and PRJNA874864, respectively. The Nanopore whole-genome assemblies of the SA accessions S1-1, S15-3, S5-10 and Cvi-0 (region Chr1-24,400,000-24,800,000) are available in PRJNA1112558 (https://dataview.ncbi.nlm.nih.gov/object/PRJNA1112558?reviewer=8tili8lhu4irrubf98lvc1nbas). Whole-genome sequencing of CRISPR lines for off-target analysis are available in PRJNA1112441 (https://dataview.ncbi.nlm.nih.gov/object/PRJNA1112441?reviewer=biuem2b3kbp7jt2eqbf5emis4i).

Research involving human participants, their data, or biological material

Policy information about studies with human participants or human data. See also policy information about sex, gender (identity/presentation), and sexual orientation and race, ethnicity and racism.

Reporting on sex and gender	n/a
Reporting on race, ethnicity, or other socially relevant groupings	n/a
Population characteristics	n/a
Recruitment	n/a
Ethics oversight	n/a

Note that full information on the approval of the study protocol must also be provided in the manuscript.

Field-specific reporting

Please select the one below that is the best fit for your research. If you are not sure, read the appropriate sections before making your selection.

☒ Life sciences ☐ Behavioural & social sciences ☐ Ecological, evolutionary & environmental sciences

For a reference copy of the document with all sections, see nature.com/documents/nr-reporting-summary-flat.pdf

Life sciences study design

All studies must disclose on these points even when the disclosure is negative.

Sample size	A sample of 83 accessions from Cape Verde Islands were chosen for WGBS. These were used to conduct GWAS, from which we identified Bonferonni-significant peaks. Major candidates were validated using mutants for which sample size was chosen to allow for significance testing for with power for effect sizes similar to those predicted from the GWAS.
Data exclusions	No data were excluded.
Replication	For the GWAS, biological replicates were conducted over SNPs rather than lines, with low frequency (<5%) SNPs (i.e., low replication SNPs) excluded from the analysis. For the RNA-seq analysis, we used biological replicates for a subset of 20/98 accessions grown, in order to verify potential gradients in the growth chambers. Biological replicates were grown at different placed across the chambers. For the WGBS data, we performed biological replicates for the fbx5 and cmt2 transgenic lines.
Randomization	Plants grown in the growth chambers were grown in a randomized block design.
Blinding	Not applicable; our phenotyping used molecular analysis. This analysis was conducted using bioinformatics pipelines, which is similar to standard blinding in a trait-based analysis.

Reporting for specific materials, systems and methods

We require information from authors about some types of materials, experimental systems and methods used in many studies. Here, indicate whether each material, system or method listed is relevant to your study. If you are not sure if a list item applies to your research, read the appropriate section before selecting a response.

Materials & experimental systems

n/a	Involved in the study
☒	☐ Antibodies
☒	☐ Eukaryotic cell lines
☒	☐ Palaeontology and archaeology
☒	☐ Animals and other organisms
☒	☐ Clinical data
☒	☐ Dual use research of concern
☐	☒ Plants

Methods

n/a	Involved in the study
☒	☐ ChIP-seq
☒	☐ Flow cytometry
☒	☐ MRI-based neuroimaging

Dual use research of concern

Policy information about dual use research of concern

Hazards

Could the accidental, deliberate or reckless misuse of agents or technologies generated in the work, or the application of information presented in the manuscript, pose a threat to:

No	Yes
☒	☐ Public health
☒	☐ National security
☒	☐ Crops and/or livestock
☒	☐ Ecosystems
☒	☐ Any other significant area

Experiments of concern

Does the work involve any of these experiments of concern:

No	Yes
☒	☐ Demonstrate how to render a vaccine ineffective
☒	☐ Confer resistance to therapeutically useful antibiotics or antiviral agents
☒	☐ Enhance the virulence of a pathogen or render a nonpathogen virulent
☒	☐ Increase transmissibility of a pathogen
☒	☐ Alter the host range of a pathogen
☒	☐ Enable evasion of diagnostic/detection modalities
☒	☐ Enable the weaponization of a biological agent or toxin
☒	☐ Any other potentially harmful combination of experiments and agents

Plants

Seed stocks	Seeds of accessions from Cape Verde and Morocco are available in our seed stock (Max Planck Institute for Plant Breeding Research, Cologne, Germany) (Brennan et al. 2014; Fulgione et al. 2022). Seeds of fbx5 T-DNA mutants from Syngenta Arabidopsis Insertion Library (SAIL_190_D02) and SALK (SALK_082977), arabidillo-2 (SAIL_162_B11), double mutant fbx5/arabidillo-2 (SAIL_190_D02/SAIL_162_B11), and FBX5 overexpressing lines were provided by Juliet Coates and are described in (Coates et al. 2006). Seeds from the 1001GP accessions Dör-10 and UKID116, the cmt2 T-DNA mutant (SAIL_906_G03), and Col-0 (CS76114) were ordered from the Nottingham Arabidopsis Stock Centre (NASC).
Novel plant genotypes	*Describe the methods by which all novel plant genotypes were produced. This includes those generated by transgenic approaches, gene editing, chemical/radiation-based mutagenesis and hybridization. For transgenic lines, describe the transformation method, the number of independent lines analyzed and the generation upon which experiments were performed. For gene-edited lines, describe the editor used, the endogenous sequence targeted for editing, the targeting guide RNA sequence (if applicable) and how the editor was applied.*
Authentication	All plants used belong to the species Arabidopsis thaliana, which was validated using whole genome sequence data. T-DNA mutant lines were validated using genotyping of insertions and CRISPR mutants were validated and checked for off-target effects using whole genome sequencing to 59.7 to 81.1x coverage.
